# Serine Phosphorylation of IRS1 Correlates with Aβ-Unrelated Memory Deficits and Elevation in Aβ Level Prior to the Onset of Memory Decline in AD

**DOI:** 10.3390/nu11081942

**Published:** 2019-08-17

**Authors:** Wei Wang, Daisuke Tanokashira, Yusuke Fukui, Megumi Maruyama, Chiemi Kuroiwa, Takashi Saito, Takaomi C. Saido, Akiko Taguchi

**Affiliations:** 1 Department of Integrative Aging Neuroscience, National Center for Geriatrics and Gerontology, Obu, Aichi 474-8511, Japan; 2 Laboratory for Proteolytic Neuroscience, RIKEN Center for Brain Science, Wako, Saitama 351-0106, Japan; 3 Department of Neurocognitive Science, Nagoya City University Graduate School of Medical Science, Nagoya, Aichi 467-8601, Japan

**Keywords:** IRS1, serine phosphorylation, hippocampus, diabetes, aging, Alzheimer’s disease, memory decline, Aβ, AMPK, energy depletion

## Abstract

The biological effects of insulin signaling are regulated by the phosphorylation of insulin receptor substrate 1 (IRS1) at serine (Ser) residues. In the brain, phosphorylation of IRS1 at specific Ser sites increases in patients with Alzheimer’s disease (AD) and its animal models. However, whether the activation of Ser sites on neural IRS1 is related to any type of memory decline remains unclear. Here, we show the modifications of IRS1 through its phosphorylation at etiology-specific Ser sites in various animal models of memory decline, such as diabetic, aged, and amyloid precursor protein (APP) knock-in ^NL-G-F^ (APPKI^NL-G-F^) mice. Substantial phosphorylation of IRS1 at specific Ser sites occurs in type 2 diabetes- or age-related memory deficits independently of amyloid-β (Aβ). Furthermore, we present the first evidence that, in APPKI^NL-G-F^ mice showing Aβ42 elevation, the increased phosphorylation of IRS1 at multiple Ser sites occurs without memory impairment. Our findings suggest that the phosphorylation of IRS1 at specific Ser sites is a potential marker of Aβ-unrelated memory deficits caused by type 2 diabetes and aging; however, in Aβ-related memory decline, the modifications of IRS1 may be a marker of early detection of Aβ42 elevation prior to the onset of memory decline in AD.

## 1. Introduction

Insulin signaling mediated by insulin receptor substrates 1 and 2 (IRS1 and IRS2) is involved in the regulation of growth, glucose homeostasis, energy metabolism, and lifespan [1,2,3,4]. The biological effects of insulin signaling are regulated by the modulation of IRS proteins through serine (Ser) and threonine (Thr) phosphorylation [5,6]. Notably, IRS1 is known to be abundantly phosphorylated at Ser and Thr residues regardless of insulin or IGF1 stimulation [5,7]. In vitro studies have demonstrated the relationship between IRS1 Ser/Thr phosphorylation and canonical downstream signaling components, including Akt/protein kinase B, glycogen synthase kinase 3 beta (GSK3β), and ribosomal protein S6 kinase (S6K) [5]. Under physiological and pathological conditions, Ser/Thr phosphorylation of IRS1 is potentially mediated by multiple kinases, including AMP-activated protein kinase (AMPK), conventional and novel protein kinase C (PKC), and c-Jun N-terminal kinases (JNKs), in response to intracellular energy status, nutritional conditions, and inflammatory stimulation [5,6,8]. 

Among the numerous Ser residues on IRS1, a few sites, including human(h)Ser312/mouse(m)Ser307, hSer616/mSer612, hSer636/mSer632, hSer639/mSer635, and hSer1101/mSer1097, have been studied because a limited number of phosphospecific antibodies were previously commercially available.

Nonetheless, hSer312/mSer307 has been widely investigated and implicated in insulin resistance under metabolic stress conditions and in inflammatory conditions such as obesity, hyperinsulinemia, and dyslipidemia [5,9,10]. However, IRS1 mSer307 knock-in mice display insulin resistance rather than increased insulin sensitivity, suggesting that IRS1 mSer307 is a positive regulatory site and is essential for normal insulin signaling [11]. IRS1 mSer612 and mSer632/635 have been cited as negative regulatory sites for IRS1 signaling through tyrosine phosphorylation [5], and mSer1097, regarded as a potential mammalian target of rapamycin (mTOR) /S6K signaling pathway, is activated in the liver of model animals of obesity [12]. However, the roles of these sites remain largely unknown because they have been examined in different context.

In the central nervous system, studies on postmortem brain tissues of patients with Alzheimer’s disease (AD) have revealed increased phosphorylation of IRS1 at hSer312/mSer307, hSer616/mSer612, hSer636/mSer632, and hSer639/mSer635 compared with that in non-AD control subjects [13,14,15,16]. The phosphorylation levels of IRS1 at hSer312/mSer307 and hSer616/mSer612 are considerably elevated in the brains of patients with AD [15]. In animal studies, multiple AD mouse models display increased phosphorylation of IRS1 at mSer307 and/or mSer632 or mSer612 [16,17,18]. However, whether the modifications of IRS1 via Ser phosphorylation are involved in memory decline in amyloid precursor protein (APP) knock-in (KI)^NL-G-F^ (APPKI^NL-G-F^) mice, a novel AD mouse model, remains unclear [19]. Meanwhile, animals with diet-induced obesity (DIO), a model of type 2 diabetes mellitus (T2DM), that were fed a 40% or 60% high-fat diet (HFD) during different periods displayed cognitive impairment accompanied by increased phosphorylation of neural IRS1 at different Ser residues (mSer1097, mSer307, or mSer612) at different ages [20,21,22]. Few studies have reported the relationship between streptozotocin (STZ)-induced type 1 diabetes-related cognitive impairment and phosphorylation of neural IRS1 at Ser sites. Similarly, in aged animals, the phosphorylation of IRS1 at mSer307 has been shown to increase in the cortex; however, whether this alteration is correlated with age-related decline in cognitive function has not been explored [23]. 

In the present study, we investigated whether the modification of hippocampal IRS1 by Ser phosphorylation is commonly associated with different types of memory decline in DIO, STZ, aged, and APPKI^NL-G-F^ mice and whether it occurs before or after the onset of memory decline in APPKI^NL-G-F^ mice. We demonstrate that the concomitant activation of specific Ser sites on hippocampal IRS1 with amyloid-β (Aβ)-unrelated memory decline occurs in DIO and aged mice, whereas STZ mice exhibit memory deficits independent of IRS1 activity. We further show that increased phosphorylation of hippocampal IRS1 at Ser sites is already observed in young APPKI^NL-G-F^ mice showing normal memory function despite increased Aβ42 level. These data suggest that the activation of Ser residues on hippocampal IRS1 is associated with non-AD-related memory impairment in T2DM and aging and with Aβ42 level, which is related to the onset of cognitive decline in AD. 

## 2. Materials and Methods 

### 2.1. Animals

C57BL/6J male mice (4 weeks of age) supplied by Japan SLC, Inc. (Shizuoka, Japan) were used to establish type 1 and 2 diabetes mellitus (T1DM, T2DM) mice and their respective control mice. Generation of high-fat-diet (HFD)-induced type 2 diabetes mice (DIO mice) was carried out as previously described [20]. Briefly, C57BL/6J mice were assigned to control wild-type (WT) mice] and HFD (DIO mice) groups, and were fed a normal diet (CE-2; CLEA Japan Inc., Tokyo, Japan) or a HFD (D12492, 60% kcal from fat; Research Diets, Inc., New Brunswick, NJ, USA) for 32 weeks, respectively. To generate Streptozotocin (STZ)-induced type 1 diabetes mice (STZ mice), eight-week-old C57BL/6J mice were intraperitoneally administered with 150 mg/kg STZ (Wako Pure Chemical Industries, Ltd., Osaka, Japan) dissolved in sodium citrate (pH 4.5) after overnight fasting, and control mice were injected with the sodium citrate buffer alone. Young and aged WT male mice, which were all pure C57BL/6J strains, were purchased from Charles River Inc., Kanagawa, Japan, or/and bred within our animal facility. APPKI^NL-G-F^ (Swedish (NL), Arctic (G), and Beyreuther/Iberian (F) mutations) homozygous mice were obtained from Dr. Saido at the Laboratory for Proteolytic Neuroscience, RIKEN Brain Science Institute, Saitama, Japan [19]. Age-matched WT mice of a similar strain (C57BL/6J) were used in the control experiments. All experiments were performed using DIO, STZ, and young and middle-aged APPKI^NL-G-F^ mice, and their respective age-matched control mice (DIO mice: 34–36 weeks, WT mice: 34–36 weeks; STZ mice: 10 weeks, WT mice: 10 weeks; young APPKI^NL-G-F^ mice: 12 weeks, WT mice: 12 weeks; middle-aged APPKI^NL-G-F^ mice: 34–36 weeks, WT mice: 34–36 weeks; young WT mice: 8–13 weeks, aged WT mice: 84 weeks). All mice were housed in a standard 12 h light–dark cycle with free access to water and food (room temperature: 25 ± 2 °C). Animal experiments were performed in compliance with the guidelines and with the approval of the ethics committee in Animal Care and Use of the National Center for Geriatrics and Gerontology in Obu, Aichi, Japan (Approval ID: 31-5).

### 2.2. Western Blotting

The protocol of western blotting used has been previously described [20]. In brief, hippocampal tissue was isolated on ice and homogenized in lysis buffer with a pellet mixer. The aliquot of hippocampal lysates was boiled for 5 min in Laemmli sodium dodecyl sulfate (SDS) sample buffer [60 mm Tris-Cl (pH 6.8), 2% sodium dodecyl sulfate, 10% glycerol, 4% β-mercaptoethanol, and 0.01% bromophenol blue]. A total of 10 μg of each SDS protein sample was loaded per lane, separated by 7.5% sodium dodecyl sulfate polyacrylamide gel electrophoresis (SDS-PAGE), and transferred to polyvinylidene difluoride membranes. Membranes were blocked using 4% Block Ace (Yukijirushi Ltd., Sapporo, Japan), incubated with the indicated primary antibodies, followed by incubation with horseradish peroxidase-conjugated secondary antibodies. Primary antibodies were rabbit anti-phospho-IRS1 [mouse Ser307/human Ser312 (mSer307/hSer312), mouse Ser612/human Ser616 (mSer612/hSer616), mouse Ser632/Ser635/human Ser636/Ser639 (mSer632/Ser635/hSer636/Ser639), mouse Ser1097/human Ser1101 (mSer1097/hSer1101)] (1:500; Cell Signaling Technology(CST)), rabbit anti-IRS1 (1:1000; CST), rabbit anti-phospho-Akt (Ser473) (1:1000; CST), rabbit anti-Akt (1:1000; CST), rabbit anti-phospho-p70S6K (Thr389) (1:1000; CST), rabbit anti-p70S6K (1:1000; CST), rabbit anti-phospho-AMPK (Thr172) (1:1000; CST), rabbit anti-AMPK (1:1000; CST), rabbit anti-phospho-GSK3β (Ser9)(1:1000; CST), rabbit anti-GSK3β (Ser9) (1:1000; CST), rabbit anti-phospho-JNK (Thr183/Tyr185) (1:1000; CST), rabbit anti-JNK (1:1000; CST), rabbit anti-phospho-aPKCζ/λ(Thr410/Thr403) (1:1000; CST), rabbit anti-aPKCζ/λ(1:1000; CST), and rabbit anti-β-tubulin (1:1000; CST). Immunodetection was performed with horseradish peroxidase-conjugated secondary antibodies (1:2000; CST) and chemiluminescence detection reagent Chemi-Lumi one L (Nacalai tesque, Kyoto, Japan) or the ImmunoStar LD (Fujifilm Wako Pure Chemical Corporation Japan, Osaka, Japan). The images were scanned using the Amersham Imager 680 (GE Healthcare UK Ltd., Little Chalfont, Buckinghamshire HP7 9NA, England ).

### 2.3. Water T Maze Test

Hippocampal-dependent spatial memory was tested using a water T maze [24,25] according to our previous report [20]. The maze consisted of a start box, a left arm, and a right arm, which was filled with water at 23 ± 1 °C up to 1 cm above the surface of the platform. Mice were allowed to swim to the right or left arm. This screening step was repeated three times at 15 s intervals. The platform was placed on the side that the mice reached less often. After the screening step, mice were allowed to explore the maze freely. If mice reached the platform, they were allowed to rest there for 5 s (correct choice). If not, the arm entry was closed with a board and they were forced to swim for 15 s as a deterrent (incorrect choice). This trial step was repeated five times at 4-min intervals. Mice were subjected to this trial step for five days. To evaluate the results of the trial, the percentage of correct responses per day was determined. After two days of rest, to test hippocampal and prefrontal cortex (PFC)-dependent working memory, the platform was moved to the opposite arm. Similarly, mice were allowed to explore the maze freely. The trial step was repeated 15 times at 4-min intervals in a day. To evaluate the results of the trial, the percentage of the correct responses were calculated after every three responses.

### 2.4. Measurement of Metabolic Parameters

Body weight and the blood glucose levels were recorded after 6 h of fasting. Blood glucose levels were measured using a portable glucose meter (ACCU-CHEK^®^ Aviva; Roche DC Japan K.K.). The level of plasma insulin at 6 h fasting was determined using an insulin enzyme-linked immunosorbent assay kit (Morinaga, Yokohama, Japan).

### 2.5. Enzyme-Linked Immunosorbent Assay (ELISA) Quantitation of Aβ

In order to measure the Aβ levels in the hippocampus, hippocampal tissue was isolated on ice and homogenized in a lysis buffer (T-PER^®^ Tissue Protein Extraction Reagent; Thermo Fisher Scientific, Waltham, MA, USA) containing a protease inhibitor cocktail (Nacalai Tesque, Kyoto, Japan) and a phosphatase inhibitor cocktail (Nacalai Tesque) with a pellet mixer. After incubation on ice for 15 min, the lysates were centrifuged at 14,200× *g* for 5 min at 4 °C and the supernatants were placed in a fresh tube. Protein concentration was determined using a Bicinchoninic acid (BCA) protein assay kit (Thermo Fisher Scientific, Waltham, MA, USA). The levels of T-PER-extractable Aβ were measured with the Human β Amyloid (1–40) enzyme-linked immunosorbent assay (ELISA) Kit Wako II (#298-64601; Fujifilm Wako Pure Chemical Corp., Osaka, Japan), the Human β Amyloid (1–42) ELISA Kit Wako, High Sensitivity (#296-64401, Fujifilm Wako Pure Chemical Corp.), the Human/Rat/Mouse β Amyloid (1–40) ELISA Kit Wako II (#294-64701; Fujifilm Wako Pure Chemical Corp., Osaka, Japan), and the Human/Rat/Mouse β Amyloid (1–42) ELISA Kit Wako, High Sensitivity (#292-64501, Fujifilm Wako Pure Chemical Corp.) in accordance with the manufacturers’ instructions.

### 2.6. Statistics

All results are presented as mean ± standard error of the mean (SEM) in the text. Statistical analyses were performed using Prism7 for Mac OS X v.7.0d (GrapPad Software Inc., La Jolla, CA, USA). Data were statistically analyzed using Student’s *t*-test and two-way analysis of variance (ANOVA). Significance is indicated as * *p* < 0.05 and ** *p* < 0.01.

## 3. Results

### 3.1. Activation of Specific Ser Residues on Hippocampal Insulin Receptor Substrate 1 (IRS1) Is Associated with T2DM-Induced Memory Impairment

**Figure 1:**
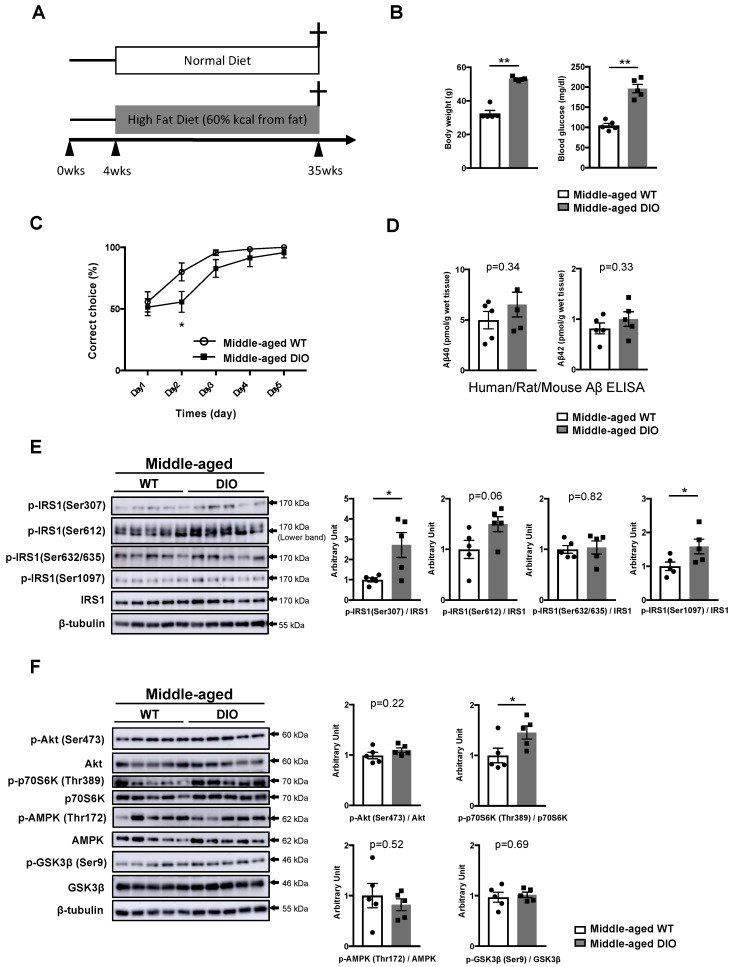
Changes in specific Ser sites on hippocampal insulin receptor substrate 1 (IRS1) in type 2 diabetes (T2DM)-induced memory impairment. (**A**) Schematic diagram of the experimental procedure. (**B**) Graphs of body weight and blood glucose level in wild-type (WT) and diet-induced obesity (DIO) mice (35 weeks of age, n = 5 mice per group). (**C**) Evaluation of learning memory function in middle-aged WT and DIO mice (n = 14 mice per group) using the water T-maze test. (**D**) Quantitative analysis of T-PER (Tissue Protein Extraction Reagent)-extractable Aβ40 and Aβ42 levels in the hippocampi of middle-aged WT and DIO mice using the human/rat/mouse β amyloid (1–40 and 1–42) enzyme-linked immunosorbent assay (ELISA) (35 weeks of age, n = 5 biologically independent samples per group). (E) Western blot analysis of phosphorylated insulin receptor substrates 1 mouse Ser307 [p-IRS1 (mSer307)], p-IRS1 (mSer612), p-IRS1 (mSer632/635), p-IRS1 (mSer1097), IRS1, and β-tubulin in the hippocampi of middle-aged WT and DIO mice (35 weeks of age, n = 5 biologically independent samples per group). Arrow indicates the p-IRS1 mSer612-corresponding band (lower band) in (**E**). Quantitative analysis of the phosphorylation of IRS1 at mSer307, mSer612, mSer632/635, and mSer1097 normalized to total protein. (**F**) Western blot analysis of phosphorylation levels of Akt Ser473, p70S6K Thr389, AMP-activated protein kinase (AMPK) Thr172, and glycogen synthase kinase 3 beta (GSK3β) Ser9 as well as total protein levels of Akt, p70 S6K, AMPK, GSK3β, and ß-tubulin in the hippocampi of middle-aged WT and DIO mice (35 weeks of age, n = 5 biologically independent samples per group). Quantitative analysis of the phosphorylation of Akt Ser473, p70S6K Thr389, AMPK Thr172, and GSK3β Ser9 normalized to the respective total protein contents. Results are presented as mean ± standard error of the mean (SEM), * *p* < 0.05; ** *p* < 0.01.

We recently reported that T2DM-related cognitive declines in 45-week-old DIO mice fed a 60% HFD for 42 weeks were accompanied by an increased phosphorylation of hippocampal IRS1 at mSer1097 [20]. While mice fed a 60% HFD for 17 days and mice or rats fed a 40% HFD for 6–8 weeks exhibited memory impairment accompanied by increased phosphorylation at hSer616/mSer612 or hSer312/mSer307 [21,22], 12-week-old DIO mice fed a 60% HFD for 9 weeks exhibited normal memory function under our experimental conditions (Figure S1), suggesting that changes in neural IRS1 Ser residues are variable on a temporal scale in DIO mice. Therefore, we investigated the phosphorylation levels of hippocampal IRS1 at Ser sites and the activities of downstream factors involved in memory impairment in 35-week-old (middle-aged) DIO mice fed a 60% HFD for 32 weeks (Figure 1A,C). At 35 weeks of age, DIO mice displayed significant weight gain, hyperglycemia, and hyperinsulinemia (Figure 1B, Figure S2A), as observed at other time points [20,21,22,26].

Furthermore, we examined whether memory impairment in middle-aged DIO mice is linked to increased levels of Aβ42, a pathological feature of AD. Biochemical analysis with specific kits (see Materials and Methods) demonstrated that, compared with age-matched wild-type (WT) mice, there was no change in Aβ40 and Aβ42 levels in the T-PER fractions obtained from the hippocampi of 35-week-old DIO mice (Figure 1D).

In contrast to DIO mice at 45 weeks of age, the phosphorylation of IRS1 at mSer307, which increases in insulin resistance, diabetes, and obesity [5], with phosphorylation at mSer1097 significantly increased in the hippocampus of DIO mice at 35 weeks of age, although there were no significant differences in phosphorylation at mSer612 and mSer632/635 between DIO and age-matched WT mice (Figure 1E, Figure S4A). Consistent with previous studies [27,28], the basal phosphorylation level of p70S6K slightly but significantly increased in the hippocampus of middle-aged DIO mice compared with that in the hippocampus of age-matched WT mice, whereas the basal phosphorylation of Akt and GSK3β and activation of AMPK and atypical protein kinase C ζ/λ (aPKC ζ/λ), downstream factors of insulin signaling, in the hippocampus were comparable between middle-aged WT and DIO mice (Figure 1F, Figures S3A, S4B and S5A). Additionally, the basal phosphorylation of JNK, an inflammation- and stress-related factor, remained unchanged in the hippocampus between the two groups (Figures S3A and S5A), although a relationship between the phosphorylation of IRS1 at mSer307 and activation of these factors in yeast cells, culture cells, and muscles has been reported [5]. These results indicate that T2DM-induced memory decline is provoked by an Aβ-independent mechanism and that the concomitant activation of IRS1 mSer307 and mSer1097 with p70S6K activation in the hippocampus is associated with memory deficits in 35-week-old DIO mice.

### 3.2. Type 1 Diabetes Mellitus (T1DM)-Induced Memory Deficits Occur Independently of IRS1 Activity

**Figure 2:**
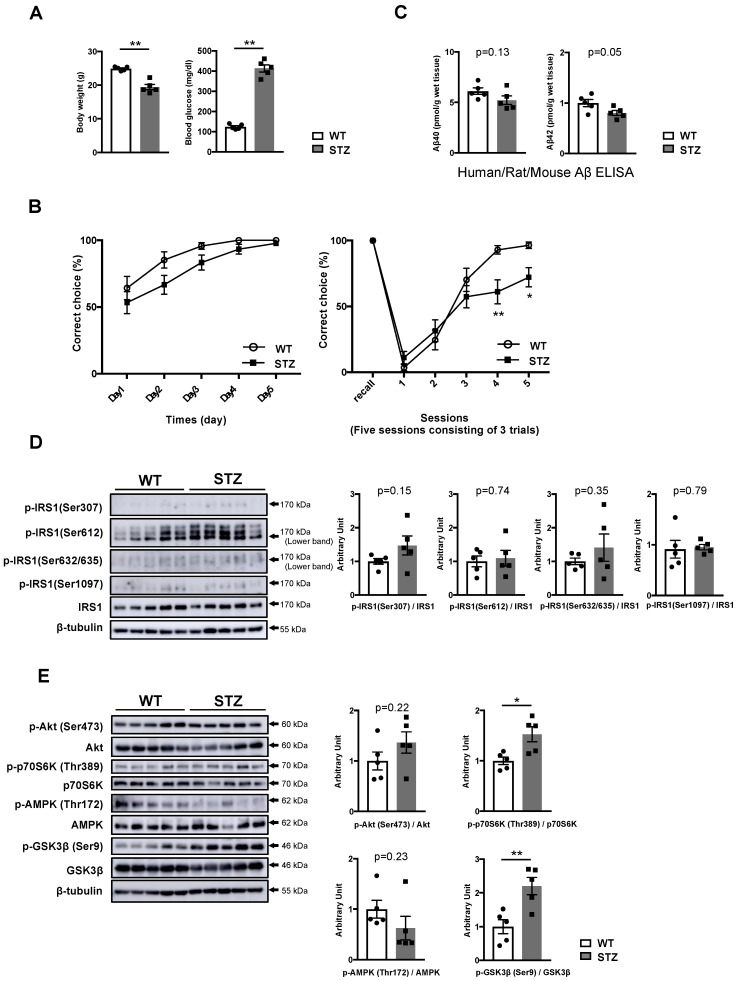
No alteration of IRS1 Ser phosphorylation in the hippocampus of T1DM mouse models. (**A**) Graphs of body weight and blood glucose level in wild-type (WT) and streptozotocin (STZ)-induced insulin-deficient type 1 diabetes (T1DM) model mice (10 weeks of age, n = 5 mice per group). (**B**) Evaluation of hippocampus-dependent learning and memory function and hippocampus/prefrontal cortex associated working memory function in WT (10 weeks of age, n = 19 mice per group) and STZ mice (10 weeks of age, n = 18 mice per group) using the water T-maze test and reverse water T-maze test. (**C**) Quantitative analysis of T-PER-extractable Aβ40 and Aβ42 levels in the hippocampi of WT and STZ mice using the human/rat/mouse β amyloid (1–40 and 1–42) ELISA (10 weeks of age, n = 5 biologically independent samples per group). (**D**) In WT and STZ-induced type 1 diabetes mice (10 weeks of age, n = 5 biologically independent samples per group), western blot analysis of phosphorylated insulin receptor substrates 1 mouse Ser307 [p-IRS1 (mSer307)], p-IRS1 (mSer612), p-IRS1 (mSer632/635), p-IRS1 (mSer1097), IRS1, and ß-tubulin was performed. Quantitative analysis of the phosphorylation of IRS1 at mSer307, mSer612, mSer632/635, and mSer1097 normalized to total protein. (**E**) Western blot analysis of phosphorylation levels of Akt Ser473, p70S6K Thr389, AMPK Thr172, and GSK3β Ser9 as well as total protein levels of Akt, p70S6K, AMPK, GSK3β, ß-tubulin in WT and STZ mice (10 weeks of age, n = 5 biologically independent samples per group). Arrows indicate the p-IRS1 mSer612-corresponding band (lower band) and the p-IRS1 mSer632/635-corresponding band (lower band) in (**D**). Quantitative analysis of the phosphorylation of Akt Ser473, p70 S6K Thr389, AMPK Thr172, and GSK3β Ser9 normalized to the respective total protein contents. Results are presented as mean ± SEM, * *p* < 0.05; ** *p* < 0.01.

To investigate whether the alteration of IRS1 through Ser phosphorylation is associated with type 1 diabetes mellitus (T1DM)-induced memory deficits, we generated STZ-induced insulin-deficient T1DM mice. Two weeks after STZ injection, STZ mice exhibited weight loss, elevated blood glucose levels (>400 mg/dL), and low insulin levels (Figure 2A, Figure S2B). While there were no significant differences in hippocampus-dependent spatial memory between WT and STZ mice, STZ mice displayed hippocampus- and prefrontal cortex-related memory decline (Figure 2B), consistent with the findings reported in previous studies [29,30,31,32]. However, STZ-induced T1DM had no effect on Aβ40 and Aβ42 levels in the T-PER fractions of the hippocampus (Figure 2C).

Subsequently, we examined the impact of STZ-induced T1DM on the phosphorylation of IRS1 at Ser residues and downstream components in the hippocampus. In STZ mice that had already developed memory impairment, the phosphorylation levels of hippocampal IRS1 at Ser residues in STZ mice were comparable to those in WT mice (Figure 2D, Figure S4C). Although the activation of p70S6K and the monotonous activities of Akt, AMPK, aPKC ζ/λ, and JNK were observed in STZ mice as well as in middle-aged DIO mice regardless of the presence or absence of Ser phosphorylation on hippocampal IRS1, the phosphorylation of GSK3β at Ser9 significantly increased in the hippocampus of STZ mice (Figure 2E, Figure S3B, S4D and S5B). These data indicate that T1DM-induced memory deficits accompanied by increased phosphorylation of GSK3β arises independently of the modification of IRS1 signaling via Ser phosphorylation and independently of Aβ elevation.

### 3.3. Phosphorylation of IRS1 at Age-Specific Ser Residues with the Activation of Downstream Kinases Is Linked to Age-Related Memory Deficits

**Figure 3:**
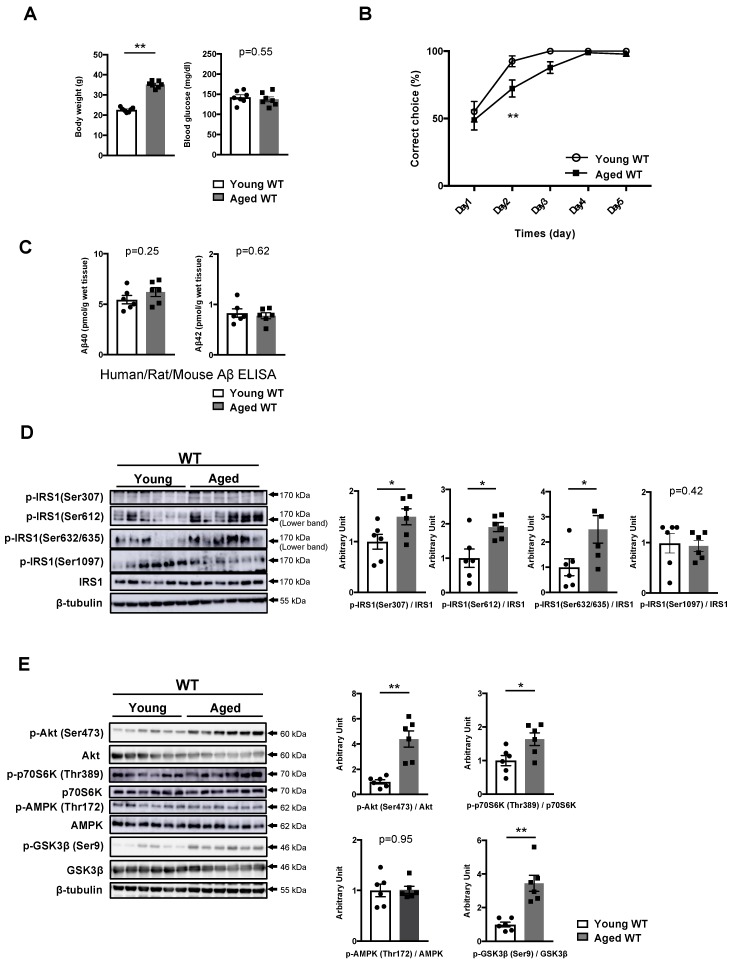
Age-related memory deficits accompanied by increased phosphorylation of IRS1 Ser sites in the hippocampus. (**A**) The graphs of body weight and blood glucose levels in young (12 weeks of age, n = 7 mice per group) and aged (84 weeks of age, n = 7 mice per group) wild-type (WT) mice. (**B**) Evaluation of learning memory functions in young WT mice (n = 16 mice per group) and aged WT mice (n = 18 mice per group) using the water T-maze test. (**C**) Quantitative analysis of T-PER-extractable Aβ40 and Aβ42 levels in the hippocampi of young (12 weeks of age, n = 6 biologically independent samples) and aged (84 weeks of age, n = 6 biologically independent samples) WT mice using the human/rat/mouse β amyloid (1–40 and 1–42) ELISA. (**D**) Western blot analysis of phosphorylated insulin receptor substrates 1 mouse Ser307 [p-IRS1 (mSer307)], p-IRS1 (mSer612), p-IRS1 (mSer632/635), p-IRS1 (mSer1097), IRS1, and ß-tubulin in the hippocampi of young WT mice (12 weeks of age, n = 6 biologically independent samples) and aged WT mice (84 weeks of age, n = 6 biologically independent samples). Quantitative analysis of the phosphorylation of IRS1 at mSer307, mSer612, mSer632/635, and mSer1097 normalized to total protein. (**E**) Western blot analysis of phosphorylation levels of Akt Ser473, p70S6K Thr389, AMPK Thr172, and GSK3β Ser9 as well as total protein levels of Akt, p70S6K, AMPK, GSK3β, and ß-tubulin in the hippocampi of young WT mice (12 weeks of age, n = 6 biologically independent samples) and aged WT mice (84 weeks of age, n = 6 biologically independent samples). Arrows indicate the p-IRS1 mSer612-corresponding band (lower band) and the p-IRS1 mSer632/635-corresponding band (lower band) in (**D**). Quantitative analysis of the phosphorylation of Akt Ser473, p70S6K Thr389, AMPK Thr172, and GSK3β Ser9 normalized to the respective total protein contents. Results are presented as mean ± SEM, * *p* < 0.05; ** *p* < 0.01.

Although memory decline associated with physiological aging can occur naturally, in contrast to pathogenic memory deficits, whether the onset processes of age-related memory impairment are related to the mechanisms underlying pathogenic memory deficits in T2DM and AD remains unclear. We examined the modification of IRS1 at Ser residues and the activity of downstream factors in the hippocampi of 21-month-old mice (aged mice). Aged mice displayed memory impairments without an increase in blood glucose and plasma insulin levels, despite an increase in body weight (Figure 3A,B, Figure S2C). Meanwhile, the Aβ40 and Aβ42 levels in the T-PER fractions of the hippocampus were comparable between young and aged WT mice (Figure 3C).

In common with DIO mice, the activation of mSer307, but not of mSer1097, on IRS1 accompanied by p70S6K activation and unchanged AMPK was observed with increased phosphorylation of IRS1 at mSer612 and mSer632/635 in the hippocampi of aged mice (Figure 3D, Figure S4E). Nonetheless, unlike in DIO mice, the basal phosphorylation levels of Akt and GSK3β prominently increased in the hippocampus of aged mice (Figure 3E, Figure S4F); however, the basal activities of aPKC ζ/λ, and JNKs remained unchanged, similar to those in DIO mice (Figure S3C and S5C). These results suggest that aging causes the concomitant phosphorylation of hippocampal IRS1 at mSer307, mSer612, and mSer632/635 with the activation of canonical downstream kinases, which may be associated with Aβ-unrelated physiological decline in memory function.

### 3.4. Increased Phosphorylation of Hippocampal IRS1 at Ser Residues in Young Amyloid Precursor Protein (APP) Knock-In (APP KI^NL-G-F^) Mice Occurs Prior to the Onset of Memory Decline

**Figure 4:**
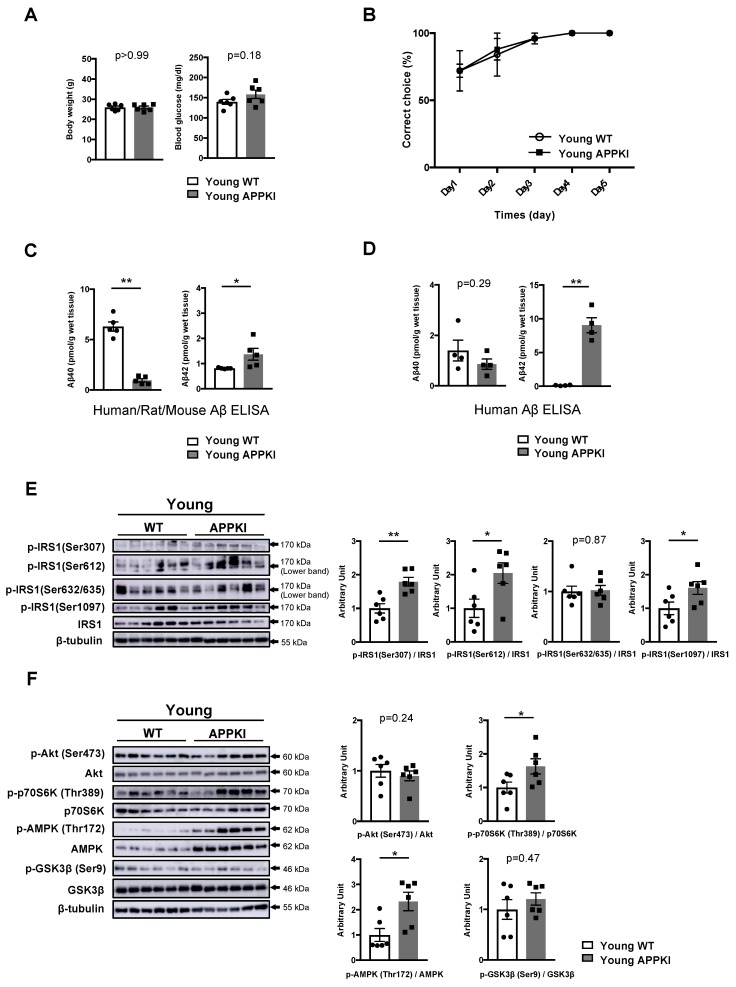
Activation of multiple Ser residues on hippocampal IRS1 unrelated to memory decline in young amyloid precursor protein (APP) knock-in (APPKI^NL-G-F^) mice. (**A**) The graphs of body weight and blood glucose levels in young wild-type (WT) and APPKI^NL-G-F^ mice (12 weeks of age, n = 6 mice per group). (**B**) Evaluation of learning memory function in young WT (n = 5 mice per group) and APP KI^NL-G-F^ mice (n = 5 mice per group) using the water T-maze test. (**C**) Quantitative analysis of T-PER-extractable Aβ40 and Aβ42 levels in the hippocampi of young WT and APPKI^NL-G-F^ mice using the human/rat/mouse β amyloid (1–40 and 1–42) ELISA (12 weeks of age, n = 5 biologically independent samples per group). (**D**) Quantitative analysis of T-PER-extractable Aβ40 and Aβ42 levels in the hippocampi of young WT and APPKI^NL-G-F^ mice using the human β amyloid (1–40 and 1–42) ELISA (12 weeks of age, n = 5 biologically independent samples per group). (**E**) Western blot analysis of phosphorylated insulin receptor substrates 1 mouse Ser307 [p-IRS1 (mSer307)], p-IRS1 (mSer612), p-IRS1 (mSer632/635), p-IRS1 (mSer1097), IRS1, and ß-tubulin in the hippocampi of young WT and APP KI^NL-G-F^ mice (12 weeks of age, n = 6 biologically independent samples per group). Arrows indicate the p-IRS1 mSer612-corresponding band (lower band) and p-IRS1 mSer632/635-corresponding band (lower band) in (**E**). Quantitative analysis of the phosphorylation of IRS1 at mSer307, mSer612, mSer632/635, and mSer1097 normalized to the respective total protein contents. (**F**) Western blot analysis of phosphorylation levels of Akt Ser473, p70S6K Thr389, AMPK Thr172, and GSK3β Ser9 as well as total protein levels of Akt, p70S6K, AMPK, GSK3β, and ß-tubulin in the hippocampi of young WT and APPKI^NL-G-F^ mice (12 weeks of age, n = 6 biologically independent samples per group). Quantitative analysis of phosphorylation of Akt Ser473, p70S6K Thr389, AMPK Thr172, and GSK3β Ser9 normalized to the respective total protein contents. Results are presented as mean ± SEM, * *p* < 0.05; ** *p* < 0.01.

To investigate whether AD-related activation of Ser residues on neural IRS1 emerges before or after the onset of memory decline, we employed a novel AD mouse model, APPKI^NL-G-F^ mice carrying a humanized Aβ sequence and three AD mutations, i.e., Swedish, Beyreuther/Iberian, and Arctic mutations, in the endogenous *App* gene [19]. First, we measured body weight and glucose metabolism in 12-week-old (young) APPKI^NL-G-F^ mice. There were no differences in terms of body weight, blood glucose level, and plasma insulin concentration between WT and APPKI^NL-G-F^ mice at 12 weeks of age (Figure 4A, Figure S2B). Next, we confirmed memory function as well as Aβ40 and Aβ42 levels in the hippocampi of young APPKI^NL-G-F^ mice. At 12 weeks of age, APPKI^NL-G-F^ mice exhibited normal memory function (Figure 4B).

**Figure 5:**
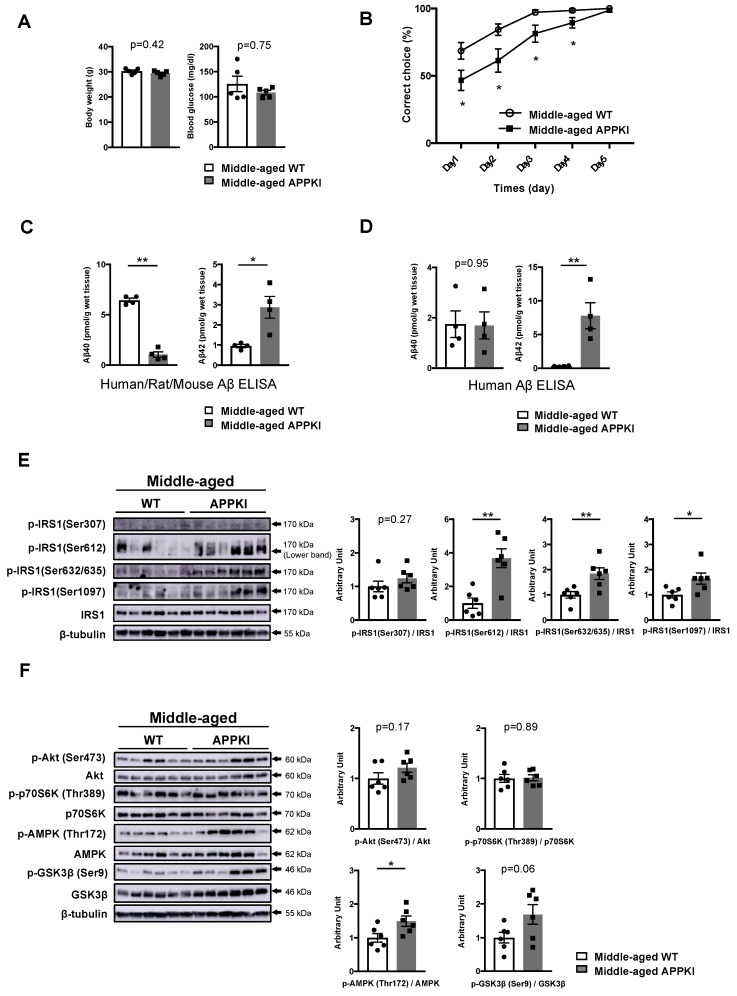
Memory decline accompanied by sustained phosphorylation of IRS1 Ser residues in the hippocampus of middle-aged APPKI^NL-G-F^ mice. (**A**) The graphs of body weight and blood glucose levels in middle-aged wild-type (WT) and APPKI^NL-G-F^ mice (34–36 weeks of age, n = 5 mice per group). (**B**) Evaluation of learning memory function in middle-aged WT (n = 14) and APPKI^NL-G-F^ mice (n = 15 mice per group) using the water T-maze test. (**C**) Quantitative analysis of T-PER-extractable Aβ40 and Aβ42 levels in the hippocampi of middle-aged WT and APPKI^NL-G-F^ mice using the human/rat/mouse β amyloid (1–40 and 1–42) ELISA (34–36 weeks of age, n = 4 biologically independent samples per group). (**D**) Quantitative analysis of T-PER-extractable Aβ40 and Aβ42 levels in the hippocampi of middle-aged WT and APPKI^NL-G-F^ mice using the human β amyloid (1–40 and 1–42) ELISA (34–36 weeks of age, n = 4 biologically independent samples per group). (**E**) Western blot analysis of phosphorylated insulin receptor substrates 1 mouse Ser307 [p-IRS1 (mSer307)], p-IRS1 (mSer612), p-IRS1 (mSer632/635), p-IRS1 (mSer1097), IRS1, and ß-tubulin in the hippocampi of middle-aged WT and APPKI^NL-G-F^ mice (34–36 weeks of age, n = 6 biologically independent samples per group). Arrow indicates the p-IRS1 mSer612 -corresponding band (lower band) in (**E**). Quantitative analysis of the phosphorylation of IRS1 at mSer307, mSer612, mSer632/635, and mSer1097 normalized to total protein. (**F**) Western blot analysis of phosphorylation levels of Akt Ser473, p70S6K Thr389, AMPK Thr172, and GSK3β Ser9 as well as total protein levels of Akt, p70S6K, AMPK, GSK3β, and ß-tubulin in the hippocampi of middle-aged WT and APPKI^NL-G-F^ mice (34–36 weeks of age, n = 6 biologically independent samples per group). Quantitative analysis of the phosphorylation of Akt Ser473, p70S6K Thr389, AMPK Thr172, and GSK3β Ser9 normalized to the respective total protein contents. Results are presented as mean ± SEM, * *p* < 0.05; ** *p* < 0.01.

Using monoclonal antibody (BNT77/BC05)-based sandwich ELISA for human/rat/mouse Aβ42, we successfully confirmed that T-PER-extractable Aβ42 level had already increased in young APP KI^NL-G-F^ mice (Figure 4C, right). A conspicuous increase in T-PER-extractable Aβ42 level in these mice was detected by human Aβ42 sandwich ELISA using monoclonal antibodies (BAN50/BC05) (Figure 4D, right). However, T-PER-extractable Aβ42 levels in both ELISA were comparable to the range of Tris-buffered saline (TBS)-extractable Aβ42 level in the brain and cortex of APPKI^NL-G-F^ mice reported by Saito et al. (2019) and Saito et al. (2014). Similarly, human/rat/mouse Aβ40 and human Aβ40 sandwich ELISA revealed the same range for Aβ40 level in the T-PER fractions of the hippocampi of young APPKI^NL-G-F^ mice (black bar on the left in Figure 4C,D and Figure 5C,D). Meanwhile, regardless of age, human/rat/mouse Aβ40 or human Aβ40 sandwich ELISA showed almost the same levels (white bar on the left in Figure 1D, Figure 2C, Figure 3C, Figure 4C and Figure 5C) or range (white bar on the left in Figure 4D and Figure 5D) of T-PER-extractable Aβ40 in WT mice, respectively.

Subsequently, we investigated the phosphorylation levels of hippocampal IRS1 at Ser residues in young APPKI^NL-G-F^ mice. Importantly, the phosphorylation of hippocampal IRS1 at mSer307, mSer612, and mSer1097, but not at mSer632/635, significantly increased in 12-week-old APPKI^NL-G-F^ mice (Figure 4E, Figure S4G) displaying normal memory function (Figure 4B). In parallel to these increases, a concomitant elevation in the basal phosphorylation of AMPK, a metabolic energy sensor, with p70S6K activation and a slight decrease in basal JNK phosphorylation were observed in these mice, along with monotonous Akt, GSK3β, and aPKC ζ/λ activity (Figure 4F, Figure S3D, S4H and S5D). Thus, in young APPKI^NL-G-F^ mice exhibiting normal memory function, the increased phosphorylation of hippocampal IRS1 at multiple Ser sites accompanied by AMPK-related low energy conditions had already occurred in the presence of increased Aβ42 level, suggesting that the elevation of Aβ42 level and/or AMPK activation provokes the activation of Ser sites on IRS1 in brains of patients with AD prior to the onset of memory decline.

### 3.5. Memory Decline in Middle-Aged APPKI^NL-G-F^ Mice Is Accompanied by the Activation of Specific Ser Sites on IRS1 and Energy Depletion

Next, we examined age-related alterations in the patterns of phosphorylation of IRS1 at Ser sites and its downstream components in the hippocampi of middle-aged APPKI^NL-G-F^ mice at 34–36 weeks of age. Consistently, body weight, blood glucose level, and plasma insulin concentration were comparable between middle-aged WT and APPKI^NL-G-F^ mice (Figure 5A, Figure S2A).

Although the onset of memory deficits was reported by 6 months in APPKI^NL-G-F^ mice [19], 6-month-old APPKI^NL-G-F^ mice did not display memory decline under our experimental conditions (data not shown). However, the water T-maze test demonstrated that middle-aged APPKI^NL-G-F^ mice exhibited memory decline after 34 weeks under our experimental conditions (Figure 5B). Meanwhile, both human/rat/mouse Aβ42 and human Aβ42 sandwich ELISA demonstrated increased levels of T-PER-extractable Aβ42 in the hippocampi of middle-aged APPKI^NL-G-F^ mice (right in Figure 5C, D) and young APPKI^NL-G-F^ mice, which were comparable to TBS-extractable Aβ42 levels in the brain and cortex of APPKI^NL-G-F^ mice [33].

While an age-related increase in the phosphorylation of IRS1 at mSer612 and mSer632/635 accompanied by sustained phosphorylation of IRS1 at T2DM-related mSer1097 site and of AMPK was observed in the hippocampi of middle-aged APPKI^NL-G-F^ mice showing memory decline (Figure 5E, Figure S4I, Table S1), there were no significant differences in phosphorylation levels at mSer307 site and of p70S6K between middle-aged WT and APPKI^NL-G-F^ mice (Figure 5E,F) owing to the age-related elevation of these factors (Figure 3D,E). Regardless of age, the activity of downstream components, such as Akt, GSK3β, aPKC ζ/λ, and JNKs, remained almost unchanged in the hippocampi of APPKI^NL-G-F^ mice (Figure 5F, Figure S3E, S4J and S5E). Our data suggest that the increased phosphorylation of hippocampal IRS1 at multiple Ser residues and persistent AMPK activation that corresponds to energy depletion accompanied by age-related elevation of Aβ42 levels are associated with the onset of memory decline in these mice.

## 4. Discussion

The present study demonstrates that memory decline in T1DM and T2DM in middle age occurs in an Aβ-independent manner, which is consistent with the findings of previous studies [34,35,36]. Similarly, age-related memory impairments arise with no alteration in Aβ levels.

In middle-aged DIO and aged mice, Aβ-unrelated memory deficits are accompanied by increased basal phosphorylation of hippocampal IRS1 at specific Ser residues, whereas Aβ-unrelated memory impairments develop in STZ mice without the modification of IRS1. Although p70S6K activation accompanied by unchanged AMPK activity in the hippocampus is mutually observed with Aβ-unrelated memory deficits in middle-aged DIO, STZ, and aged mice regardless of the presence or absence of the phosphorylation of IRS1 at Ser residues, different alterations in other downstream components were observed in these mouse models. Furthermore, we found that, in APPKI^NL-G-F^ mice, a concomitant increase in the basal phosphorylation of hippocampal IRS1 at multiple Ser sites with the activation of AMPK and elevation of Aβ42 level had already arisen at a young age, before the onset of memory decline, and that memory decline in middle age was accompanied by the persistence of these changes with age-related increase in the phosphorylation of IRS1 at specific Ser sites and in Aβ42 level.

HFD and genetically obese animals exhibit increased phosphorylation at mSer307 and p70S6K-induced phosphorylation at mSer1097 in peripheral tissues [37,38]. In the central nervous system, the involvement of non-activated AMPK with/without phosphorylation of IRS1 at mSer307 accompanied by p70S6K activation or different combinations of alterations in Akt and GSK3β activity (i.e., increased or decreased phosphorylation) in cognitive impairment in 40–45% HFD-fed and STZ mice has been reported [21,22,27,28,32,34,39,40], whereas the monotonous levels of phosphorylation of IRS1 at Ser sites and of downstream kinases including Akt, AMPK, GSK3β, and p70S6K are observed in 45% HFD-induced cognitive deficits [41]. Consistent with these results, memory decline in middle-aged DIO mice is accompanied by a concomitant increase in the basal phosphorylation of IRS1 at mSer307 and mSer1097 with p70S6K activation and monotonous activity of Akt, GSK3β, and AMPK in the hippocampus. On the other hand, in aged mice, the concomitant activation of Akt and GSK3β with an increase in the basal phosphorylation of hippocampal IRS1 at mSer307, mSer612, and mSer632/635 with p70S6K activation is observed, which is consistent with previous studies showing that the increased phosphorylation of mSer632/635 or mSer1097 on IRS1 is associated with p70S6K [5,37,38]. Given that the activation of mSer612 on IRS1 may negatively correlate with intracellular signaling and that the activation of Ser632/635 on IRS1 may occur independently of Akt [5], it is likely that, in aged mice, the increased phosphorylation of IRS1 at mSer307, mSer612, and mSer632/635 with the activation of multiple downstream factors other than AMPK may arise through reciprocal feedback regulation.

Additionally, HFD- and STZ-induced diabetes increases the phosphorylation of JNK accompanied by the activation of mSer307 on IRS1 in the brain [21,32]. The activity of JNK is elevated in aged mice and transgenic mouse models of AD, although the involvement of phosphorylation of IRS1 at Ser sites in the altered activation of JNK has not been reported [16,42]. Furthermore, the activation of aPKC ζ/λ is associated with the phosphorylation of IRS1 at Ser sites, including mSer1097 [5,20]. However, in all types of mouse models of memory impairment in the present study, the basal phosphorylation levels of JNK and aPKC ζ/λ consistently remained unchanged under our experimental conditions. These discrepancies may be due to the differences in observation time, animal species, and HFD or STZ protocols, such as age, duration of exposure, diet fat content, and drug dosages. Taken together, these results suggest that the reciprocal effects of the phosphorylation of IRS1 at T2DM- or age-related Ser sites and downstream components via feedback loops that may lead to the common alterations in the activity of p70S6K and AMPK are involved in Aβ-unrelated memory decline; however, the modification of IRS1 through Ser sites is not required for the onset of memory deficits in STZ-induced T1DM mice.

Although a link between the phosphorylation of neural IRS1 at mSer307, mSer612, and mSer632/635 and brain insulin resistance has been proposed [14,16,43], brain insulin resistance is not yet defined, and the phosphorylation of IRS1 at Ser sites emerges in insulin-dependent and insulin-independent manners [5,6]. Given that diabetes- and obesity-induced memory impairments were accompanied by the phosphorylation of IRS1 at mSer307 [5], we found that young APPKI^NL-G-F^ mice showing normal metabolism and memory function displayed increased phosphorylation of hippocampal IRS1 at three Ser residues including mSer307, mSer612, and mSer1097, in which the Aβ42 level had already increased before the onset of memory decline. Consistent with previous studies [13,14,15,16], the increased phosphorylation at mSer612 and mSer 632/635 and persistent activation of mSer1097 are observed in the hippocampus of middle-aged APPKI^NL-G-F^ mice exhibiting memory decline with age-associated increase in the Aβ42 level.

Interestingly, in both young and middle-aged APPKI^NL-G-F^ mice, the concomitant phosphorylation of hippocampal IRS1 at mSer612 and mSer1097 with the activation of AMPK is constantly observed regardless of the presence or absence of the activation of mSer307 or mSer 632/635, suggesting that persistent phosphorylation of mSer612 and mSer1097 accompanied by AMPK activation may contribute to memory dysfunction in AD.

Reportedly, the Arctic mutation in APPKI^NL-G-F^ mice reduces immunoreactivity in ELISA because the location of this mutation on the Aβ sequences is overlapped with the binding region of monoclonal antibody to Aβ used in human/rat/mouse Aβ sandwich ELISA [19]; however, T-PER-extractable Aβ40 and Aβ42 levels were successfully determined by human/rat/mouse Aβ sandwich ELISA using BNT77 (binds to the location of the Arctic mutation) and human Aβ sandwich ELISA using BAN50 (does not bind to the location of the Arctic mutation). We confirmed that the elevation of Aβ42 level is evident in young APPKI^NL-G-F^ mice and that the Aβ42 level increased in middle-aged APPKI^NL-G-F^ mice. Owing to the elevation of Aβ42 level in the hippocampi of APPKI^NL-G-F^ mice from a young age, it is likely that the increased phosphorylation of IRS1 at Ser sites is associated with Aβ42 level but not with memory decline. Consistent with this finding, a previous study showed that the phosphorylation of IRS1 at mSer307/hSer312 and mSer612/hSer616 increased in cultured hippocampal neurons exposed to Aβ oligomers prepared from synthetic Aβ1–42 peptide and in the AβO-injected hippocampi of non-human primates [16]. Interestingly, the increased basal phosphorylation of hippocampal IRS1 at multiple Ser sites in APPKI^NL-G-F^ mice is accompanied by AMPK activation regardless of the presence or absence of memory decline. Consistent with our findings, recent studies suggest that AMPK activation is implicated in brain aging and development of neurodegenerative diseases, including AD [44,45]. These results suggest that the elevation of Aβ42 accompanied by AMPK activation induced by energy depletion that occurs from a young age before the onset of memory decline is associated with increased phosphorylation of hippocampal IRS1 at multiple Ser sites and that sustained activation of these factors contributes to the onset of memory decline in middle-aged APPKI^NL-G-F^ mice.

In summary, whether the modification of IRS1 through its phosphorylation at Ser sites in the hippocampus has a pathogenic or an adaptive function remains unknown because the phosphorylation of hippocampal IRS1 at mSer307, mSer612, and mSer1097 increased when metformin improves memory deficits in middle-aged DIO mice [20,46]. Studies using mutant mice with IRS1 Ser residues in the brain will help us to understand the roles of Ser sites in memory function and to identify unrecognized downstream pathways.

## 5. Conclusions

Our findings indicate that the phosphorylation of IRS1 at disease-specific Ser residues in the hippocampus may be a potential marker of Aβ-unrelated memory impairments induced by T2DM and aging. Alternatively, in Aβ-related memory decline in AD, the modification of IRS1 via its phosphorylation at multiple Ser sites accompanied by the activation of AMPK, a sensor of energy metabolism, may be a marker of the response to an early detection of elevated Aβ42 level before the onset of memory decline in AD.

## Supplementary Materials

The following are available online at https://www.mdpi.com/2072-6643/11/8/1942/s1: Figure S1: No difference in memory function between wild-type (WT) and DIO mice at a young age.; Figure S2: Levels of blood insulin in the respective mouse models.; Figure S3: Evaluation of the other signaling factors associated with IRS1 signaling in the hippocampus.; Figures S4 and S5: Full images of Western blots; Table S1: Summary of phosphorylated Ser residues on hippocampal IRS1 in all models.
