# Supplementary material for: Serine Phosphorylation of IRS1 Correlates with Aβ-Unrelated Memory Deficits and Elevation in Aβ Level Prior to the Onset of Memory Decline in AD
Source: Nutrients. 2019 Aug 17;11(8):1942. doi: 10.3390/nu11081942 (PMC6723493; doi:10.3390/nu11081942)
Supplement: Supplementary file 1 [file nutrients-11-01942-s001.pdf]

Supplementary File 1 (Figure S1)

A

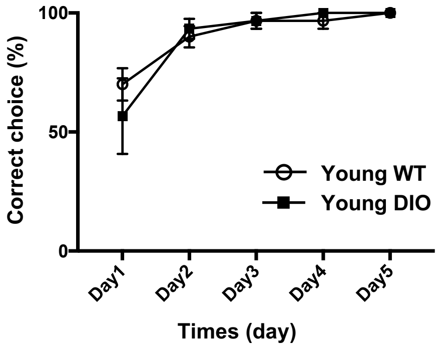

Supplementary File 1 (Figure S1) No difference in memory function between wild-type (WT) and DIO mice at a young age. (A) The water T-maze test for assessing learning and memory function in young WT and DIO mice (12 weeks of age, n = 6 mice per group).

Supplementary File 2 (Figure S2)

A

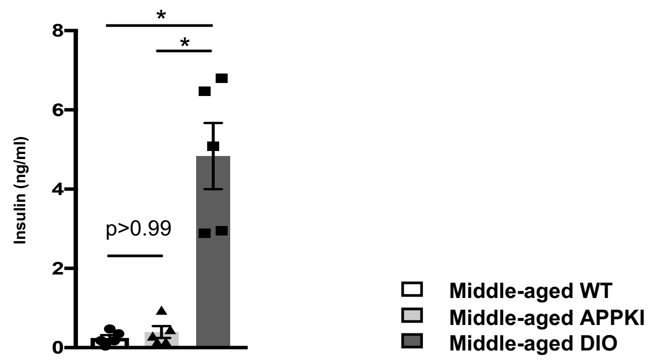

B

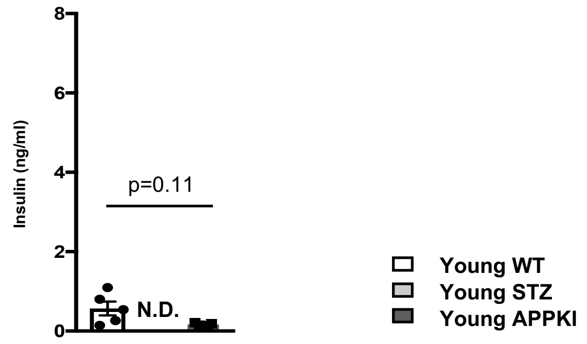

C

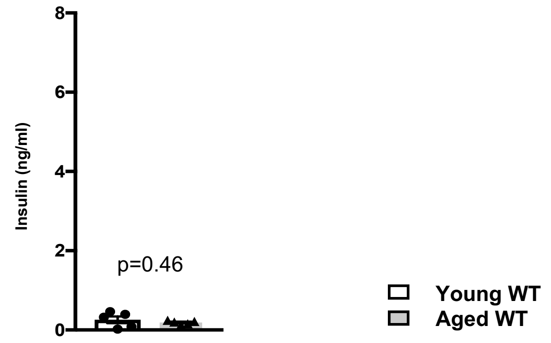

Supplementary File 2 (Figure S2). Levels of blood insulin in the respective mouse models. (A) Quantitative comparison of 3-6 h fasting blood insulin levels of middle-aged wild type (WT) (34–36 weeks of age, n = 5 mice per group), $APPKI^{NL\text{-}G\text{-}F}$ (34–36 weeks of age, n = 5 mice per group), and DIO mice (34–36 weeks of age, n = 5 mice per group). (B) Quantitative comparison of 3-6 h fasting blood insulin levels of young WT (10 weeks of age, n = 5 mice per group), STZ (10 weeks of age, n = 4 mice per group), and $APPKI^{NL\text{-}G\text{-}F}$ mice (12 weeks of age, n = 5 mice per group). (C) Quantitative comparison of 3-6 h fasting blood insulin levels of young (12 weeks of age, n = 5 mice per group) and aged (84 weeks of age, n = 5 mice per group) WT mice. Results are presented as mean ± SEM, * $p < 0.05$; ** $p < 0.01$.

Supplementary File 3 (Figure S3)

A

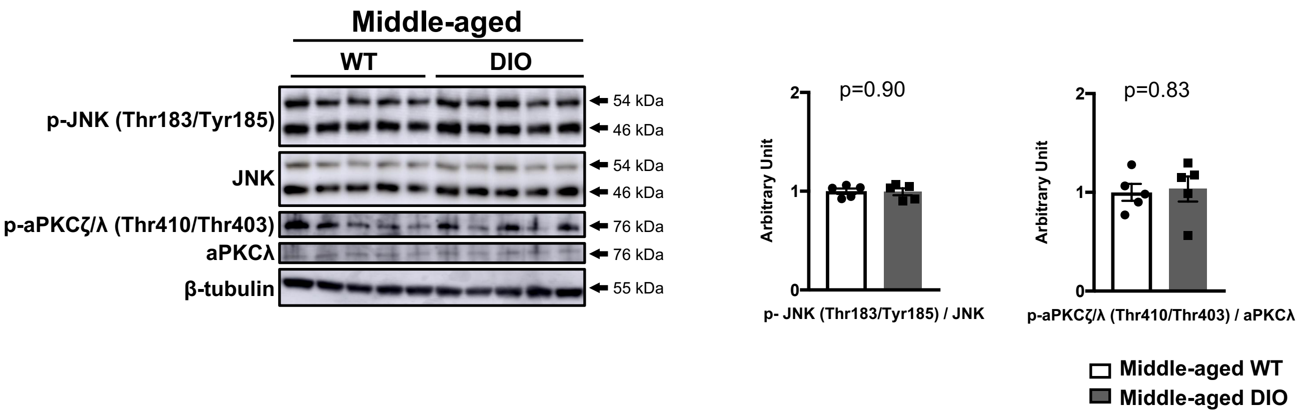

B

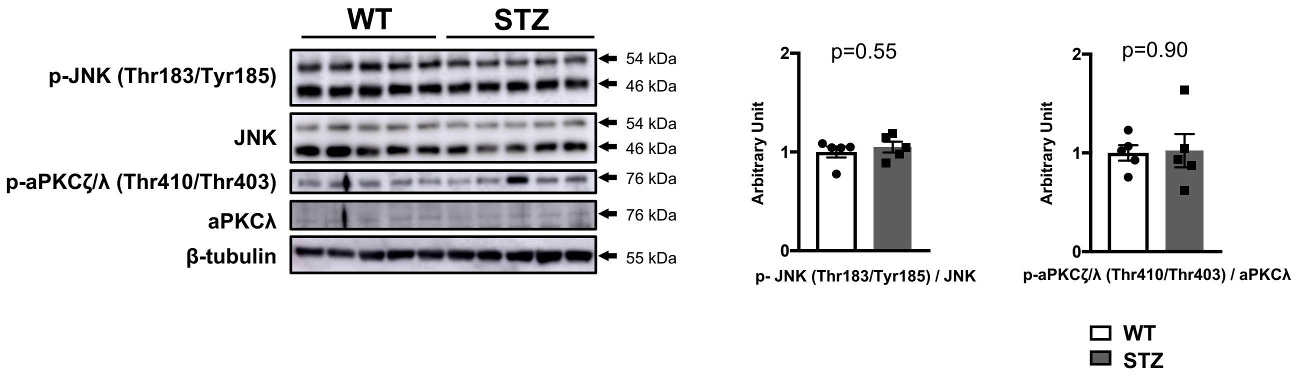

C

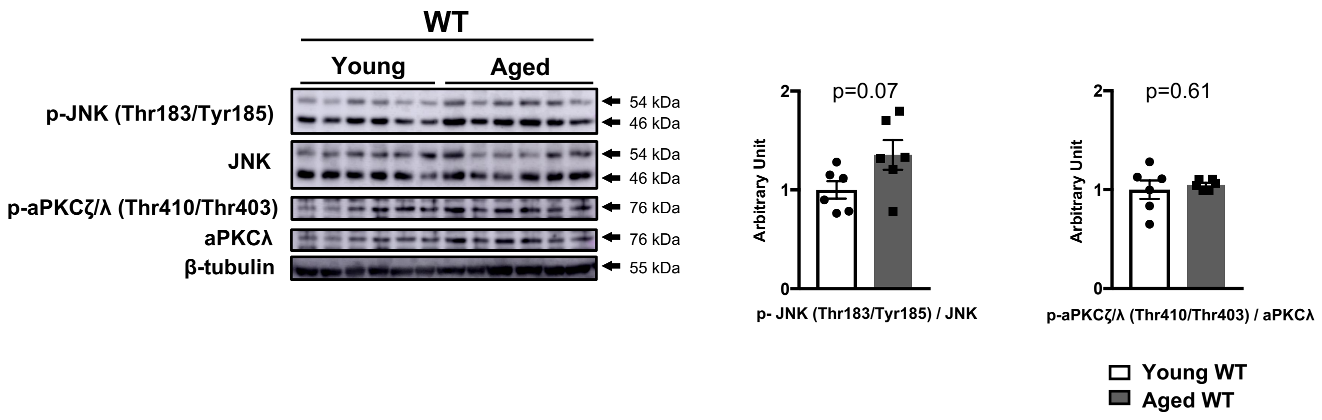

Supplementary File 3 (Figure S3) (continued)

D

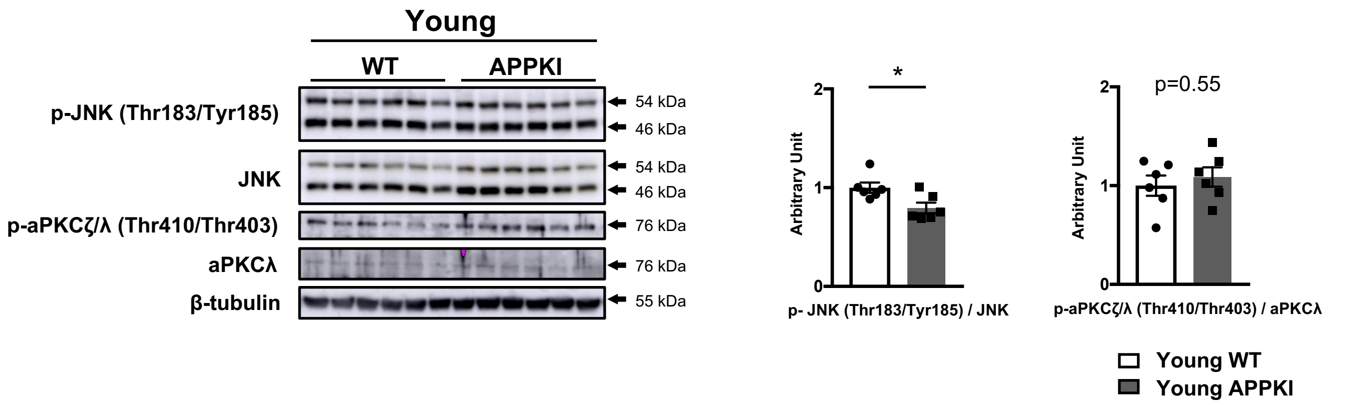

E

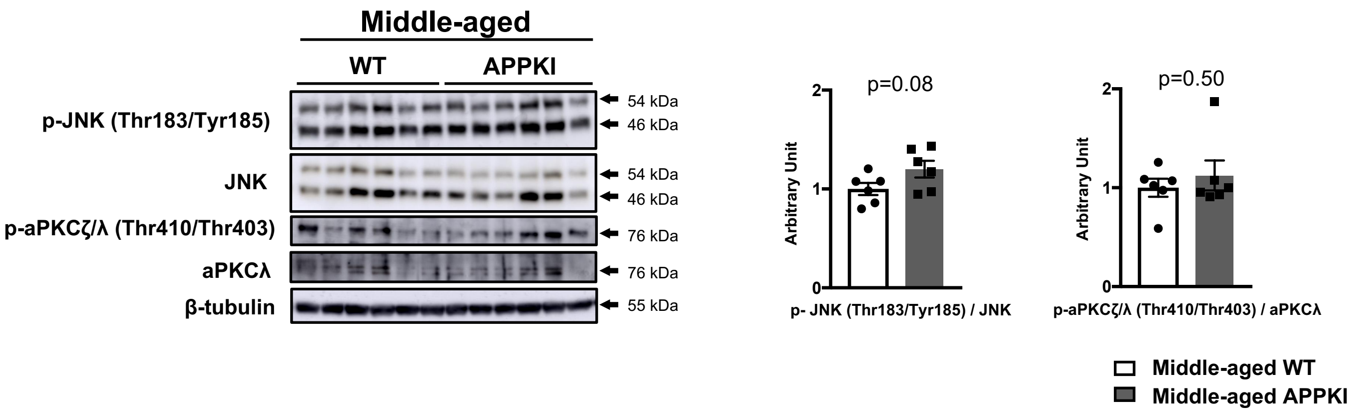

Supplementary File 3 (Figure S3). Evaluation of the other signaling factors associated with IRS1 signaling in the hippocampus. (A-E) Western blot analysis of phosphorylation levels of JNK Thr183/Tyr185, and aPKCζ/λ Thr410/Thr403 as well as total protein levels of JNK, aPKCλ, and β-tubulin in the hippocampi of middle-aged wild-type (WT) and DIO mice (35 weeks of age, n = 5 biologically independent samples per group) [A], WT and STZ mice (10 weeks of age, n = 4 biologically independent samples per group) [B], young (12 weeks of age, n = 6 biologically independent samples) and aged (84 weeks of age, n = 6 biologically independent samples) WT mice [C], young WT and APPKI$^{NL\text{-}G\text{-}F}$ mice (12 weeks of age, n = 6 biologically independent samples per group) [D], and middle-aged WT and APPKI$^{NL\text{-}G\text{-}F}$ mice (34–36 weeks of age, n = 6 biologically independent samples per group) [E]. Quantitative analysis of phosphorylation levels of JNK Thr183/Tyr185, and aPKCζ/λ Thr410/Thr403 normalized to the respective total protein contents in (A-E). Results are presented as mean ± SEM, * $p < 0.05$; ** $p < 0.01$.

Supplementary File 4 (Figure S4)

A Figure 1E

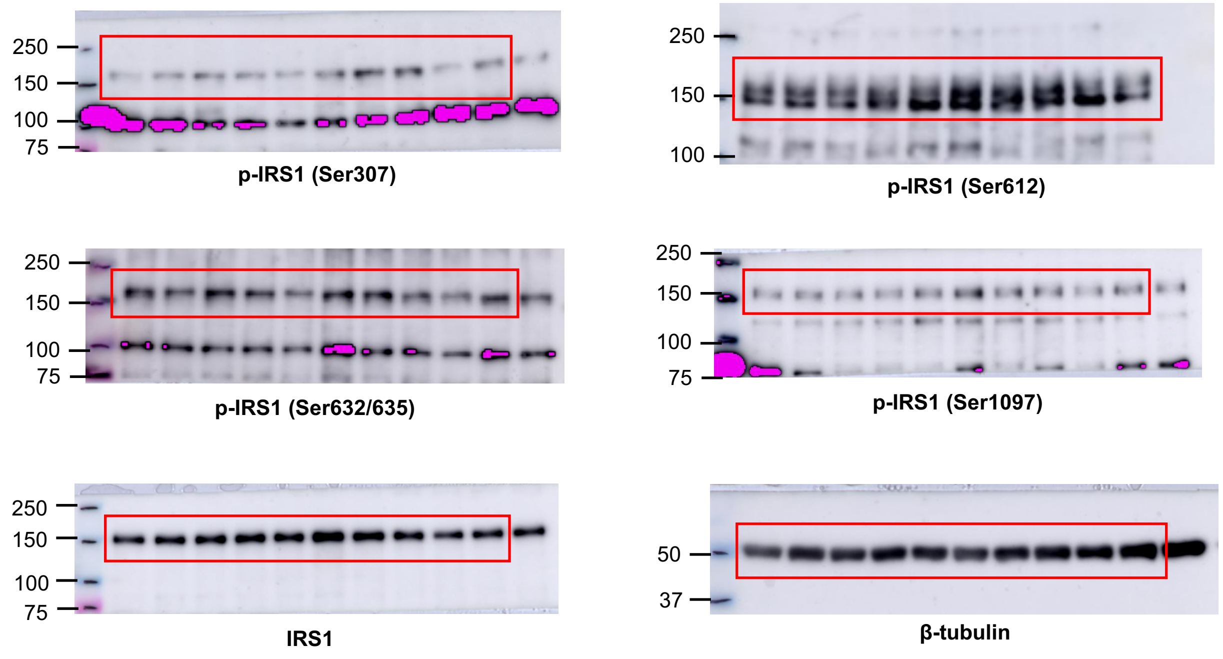

B Figure 1F

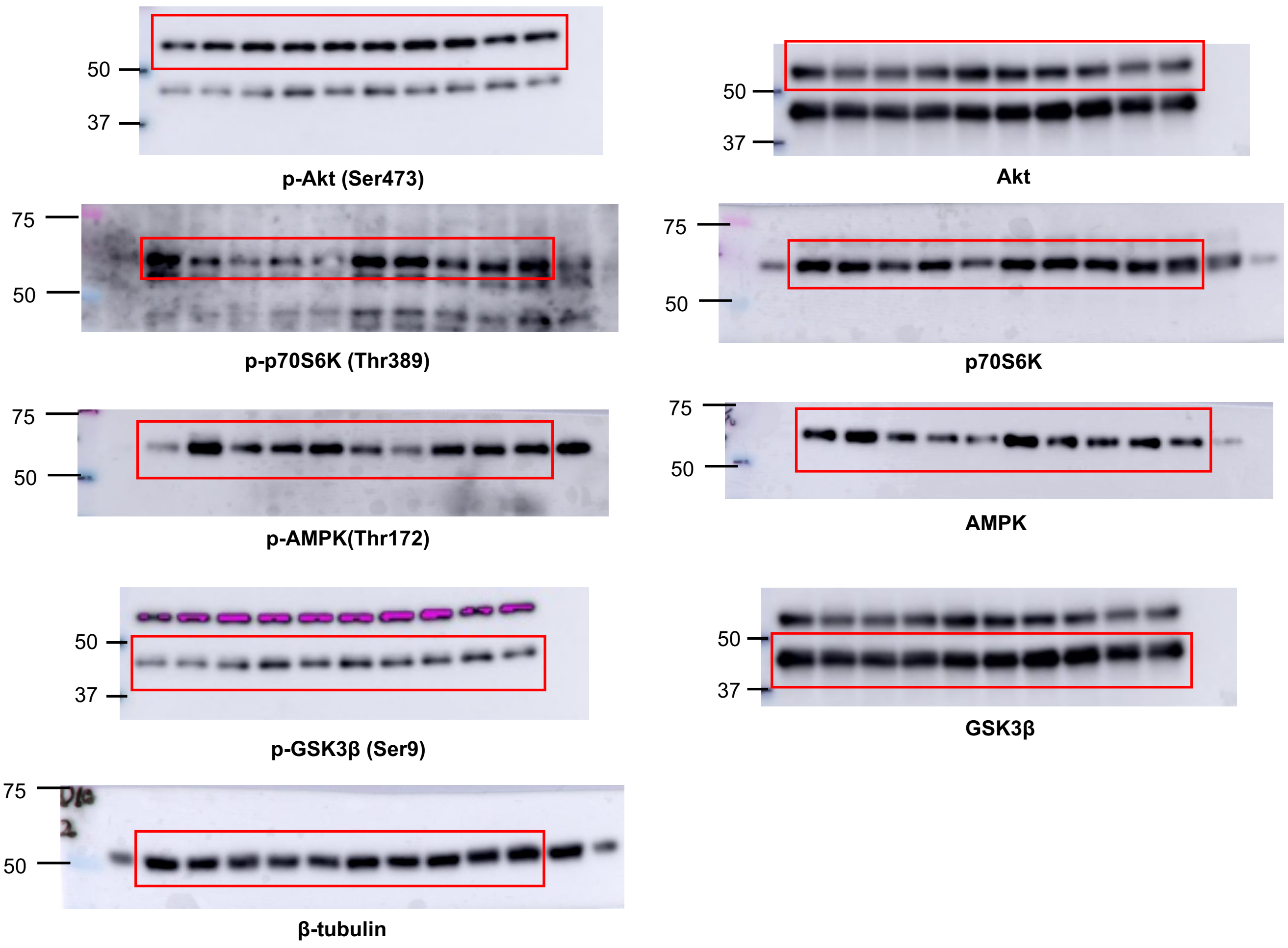

Supplementary File 4 (Figure S4) (continued)

C Figure 2D

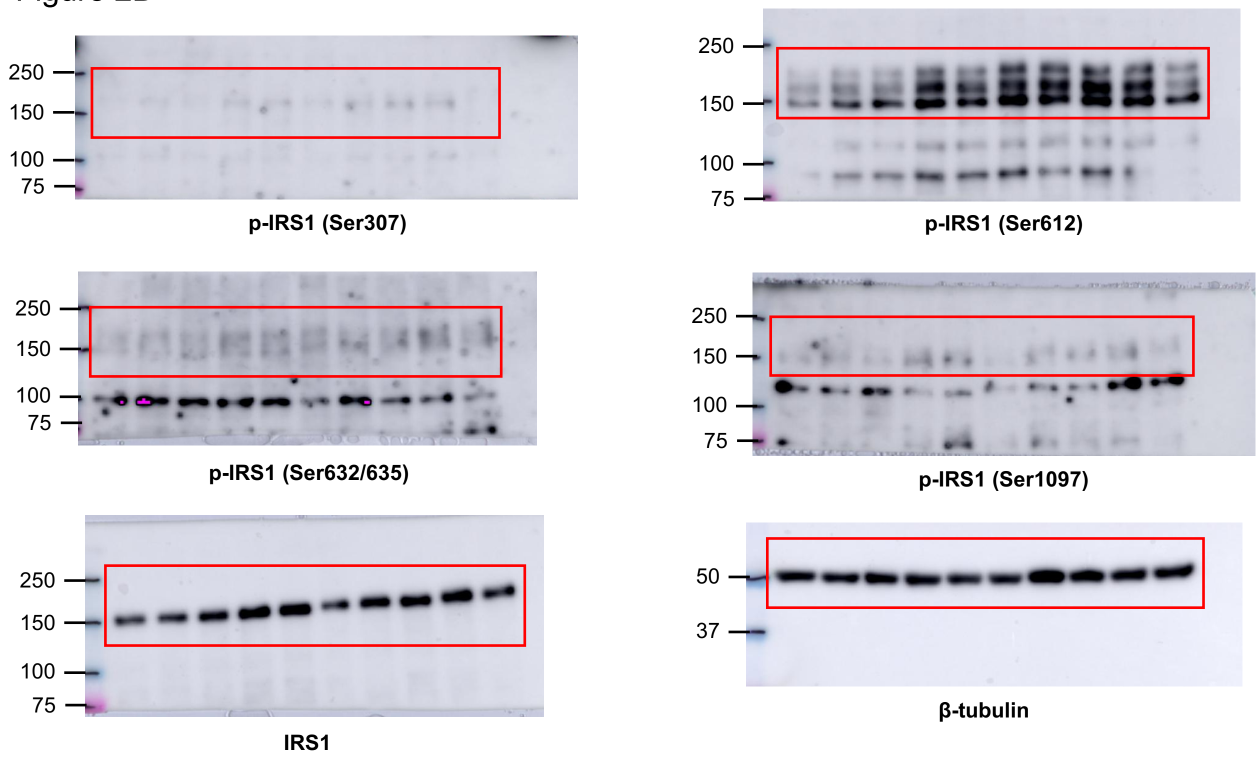

D Figure 2E

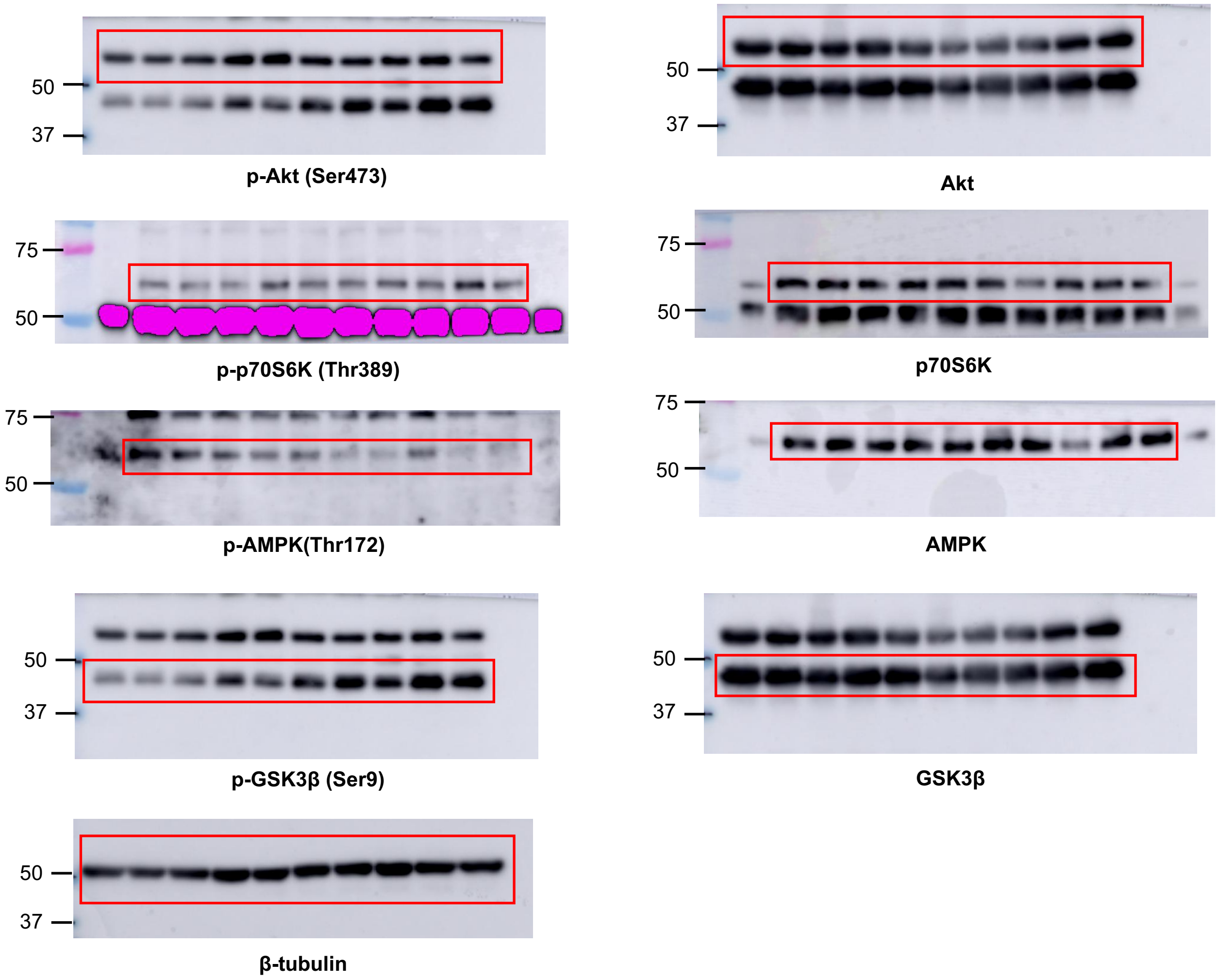

Supplementary File 4 (Figure S4) (continued)

E Figure 3D

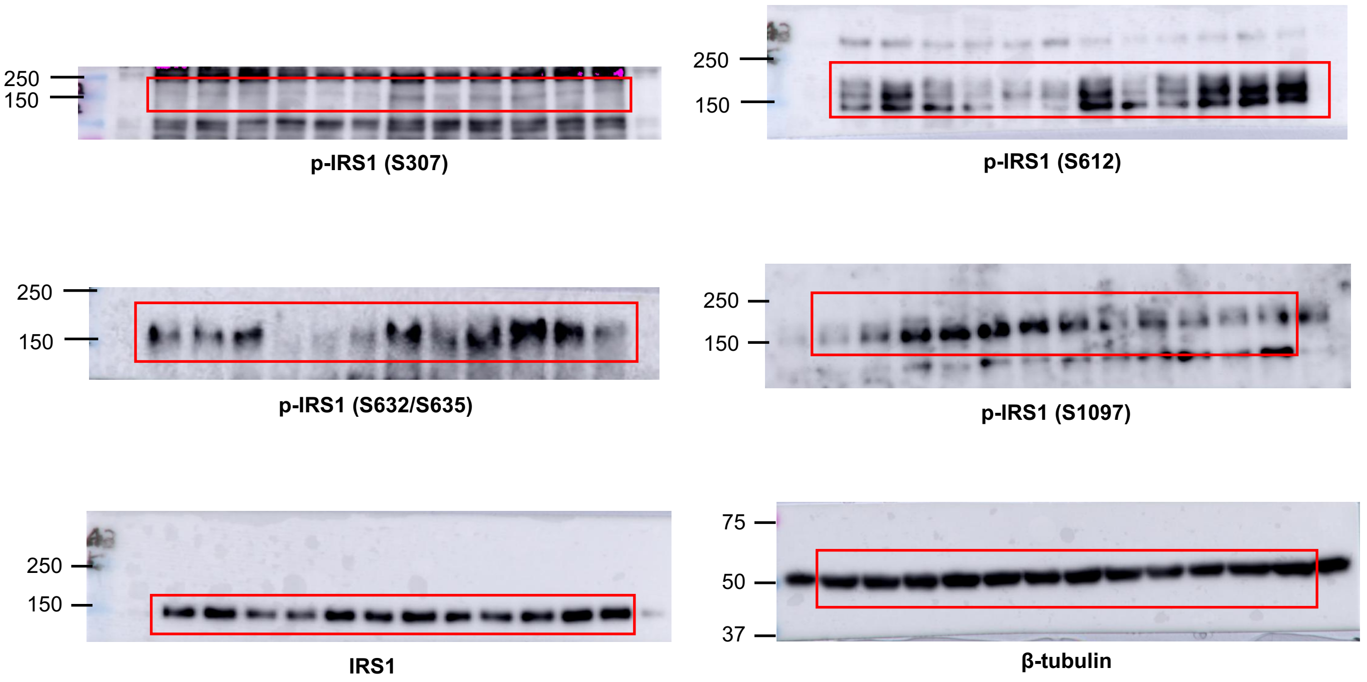

F Figure 3E

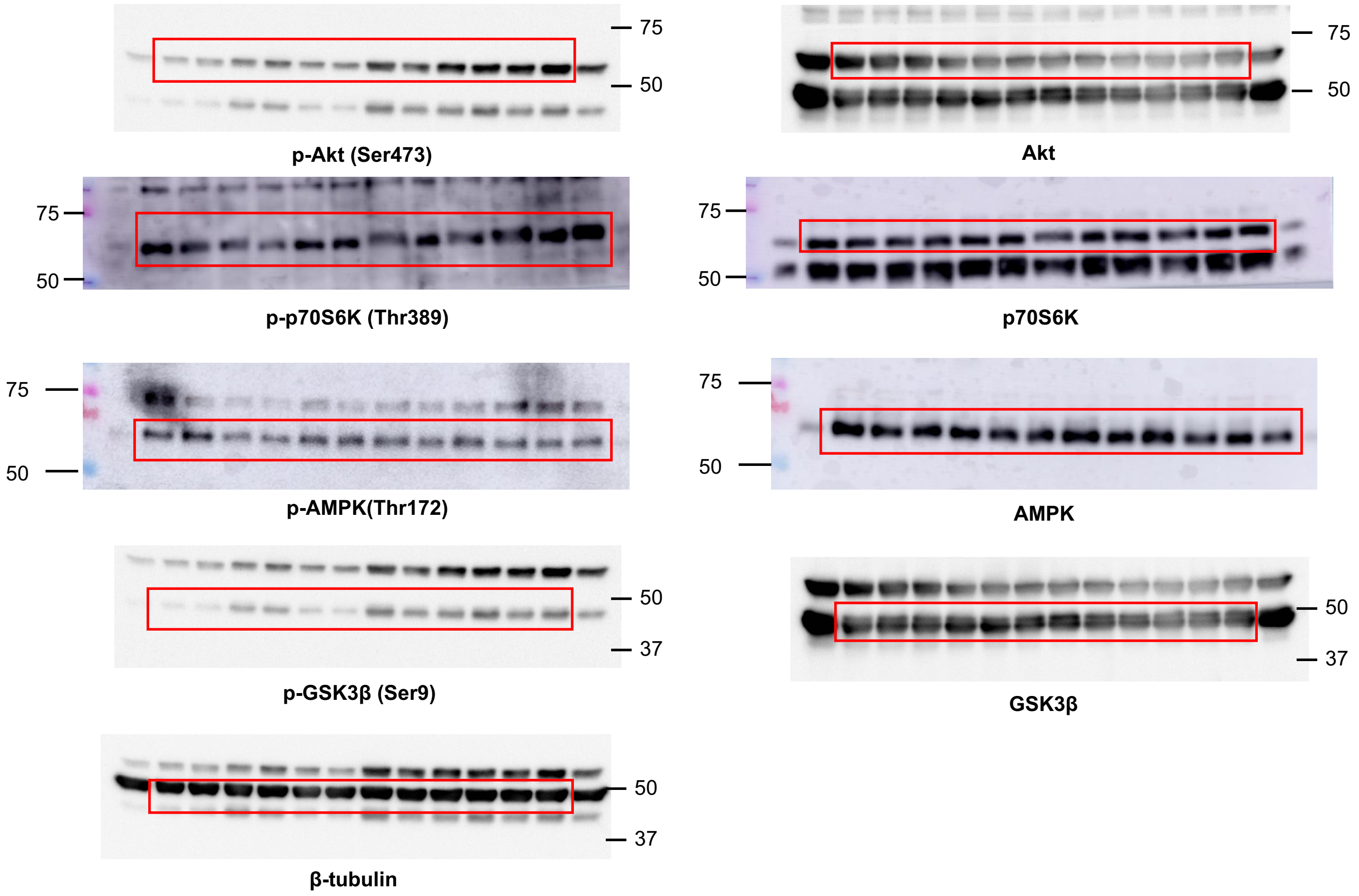

Supplementary File 4 (Figure S4) (continued)

G Figure 4E

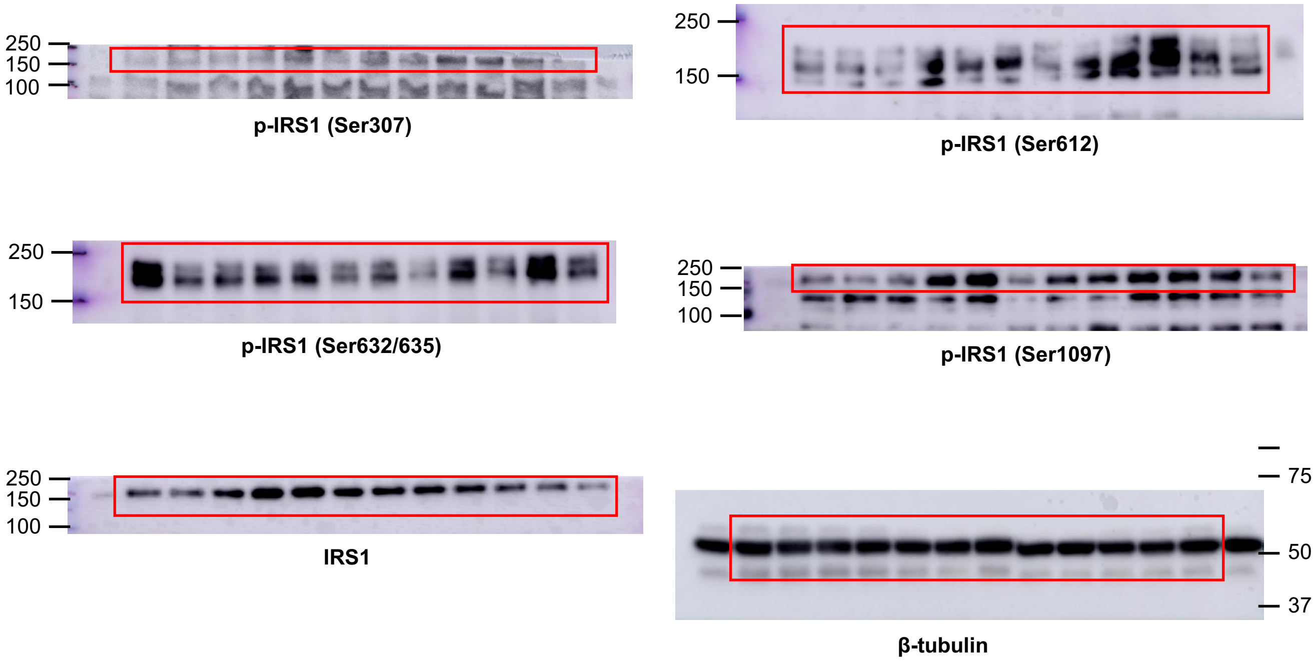

H Figure 4F

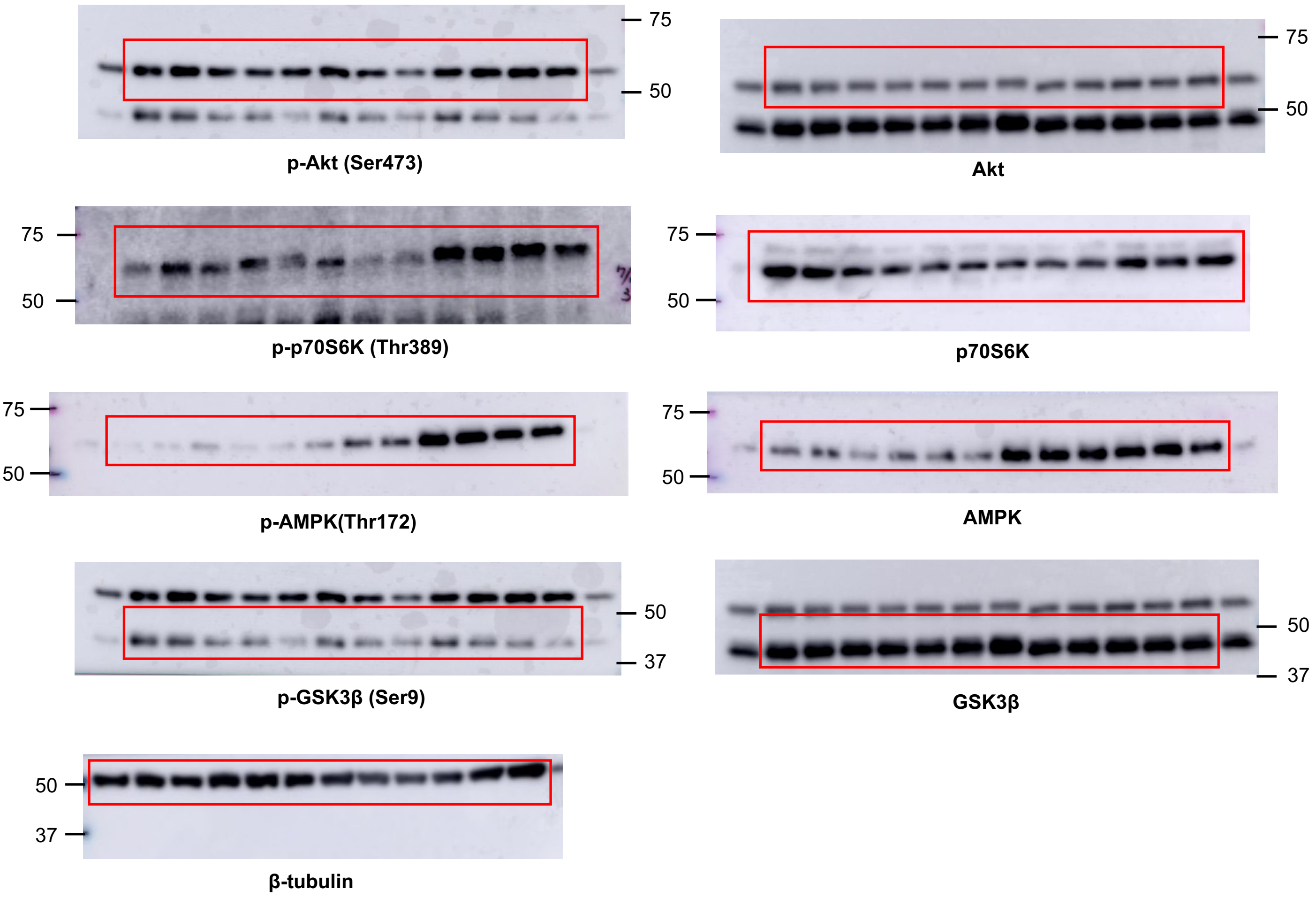

Supplementary File 4 (Figure S4) (continued)

I Figure 5E

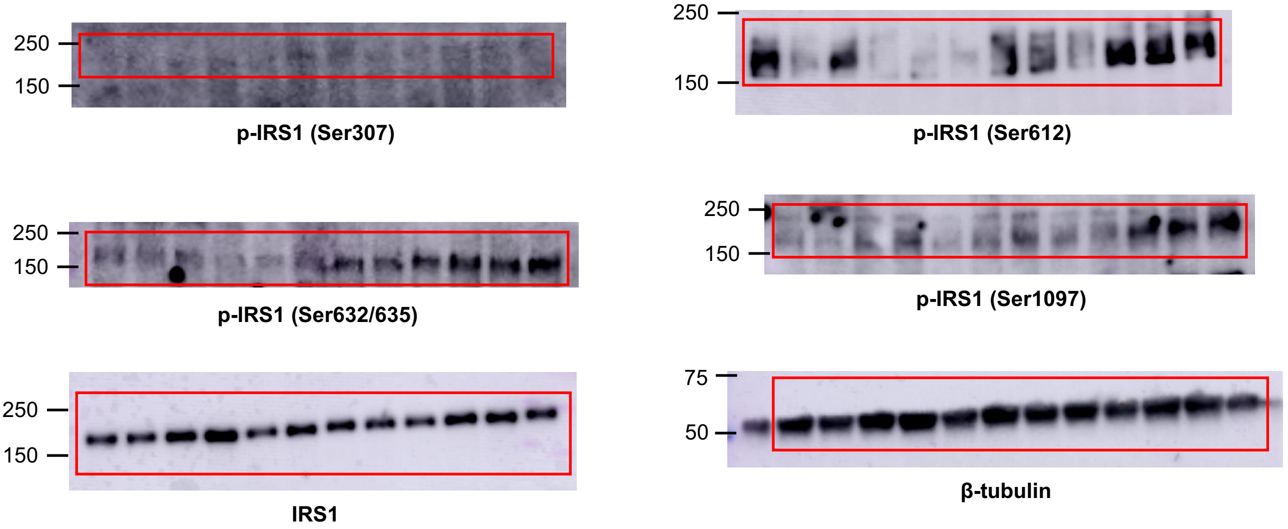

J Figure 5F

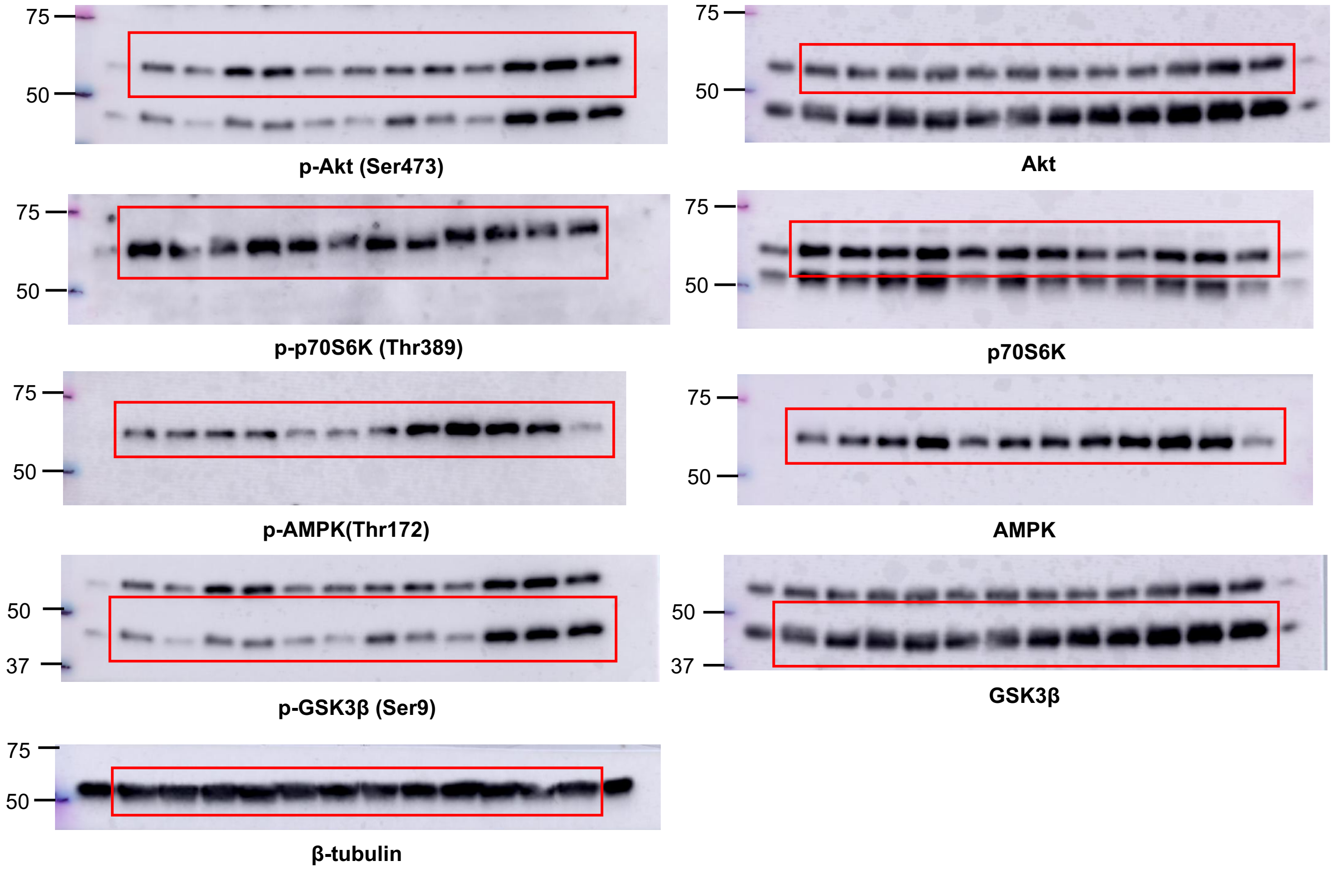

Supplementary File 4 (Figure S4). Full images of western blots. (A) Figure 1E; (C) Figure 2D; (E) Figure 3D; (G) Figure 4E; (I) Figure 5E: phosphorylated-insulin receptor substrates 1 mouse Ser307 [p-IRS1 (mSer307)], p-IRS1 (mSer612), p-IRS1 (mSer632/635), p-IRS1 (mSer1097), IRS1, and β-tubulin. (B) Figure 1F; (D) Figure 2E; (F) Figure 3E; (H) Figure 4F; (J) Figure 5F: phosphorylated and total Akt, p70S6K, AMPK, GSK3β, and β-tubulin.

Supplementary File 5 (Figure S5)

A Figure S3A

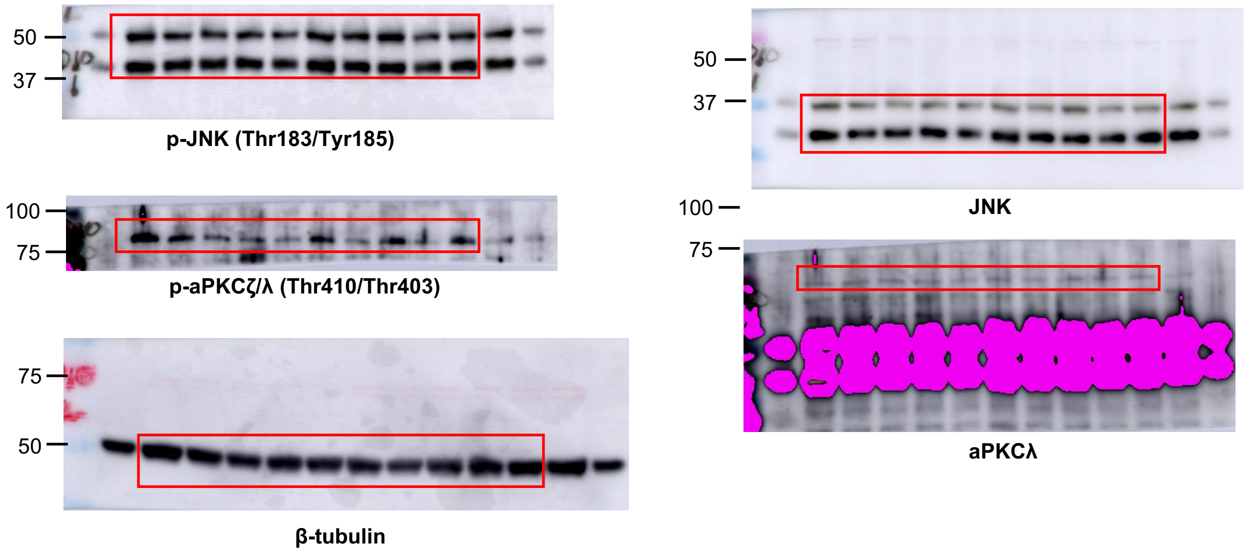

B Figure S3B

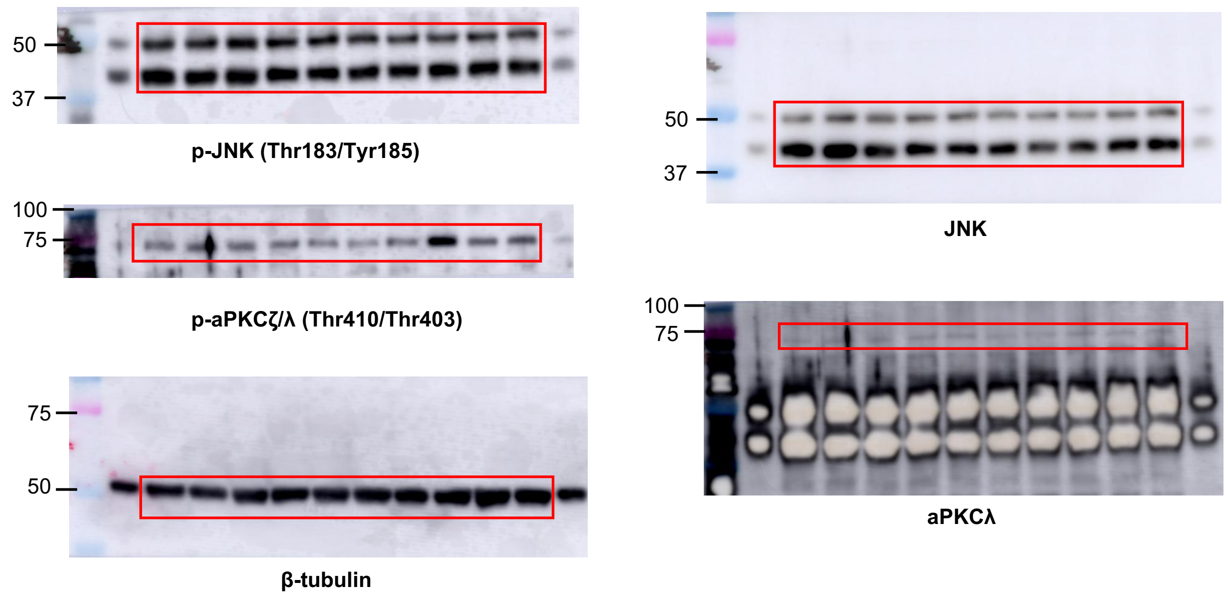

Supplementary File 5 (Figure S5) (continued)

C Figure S3C

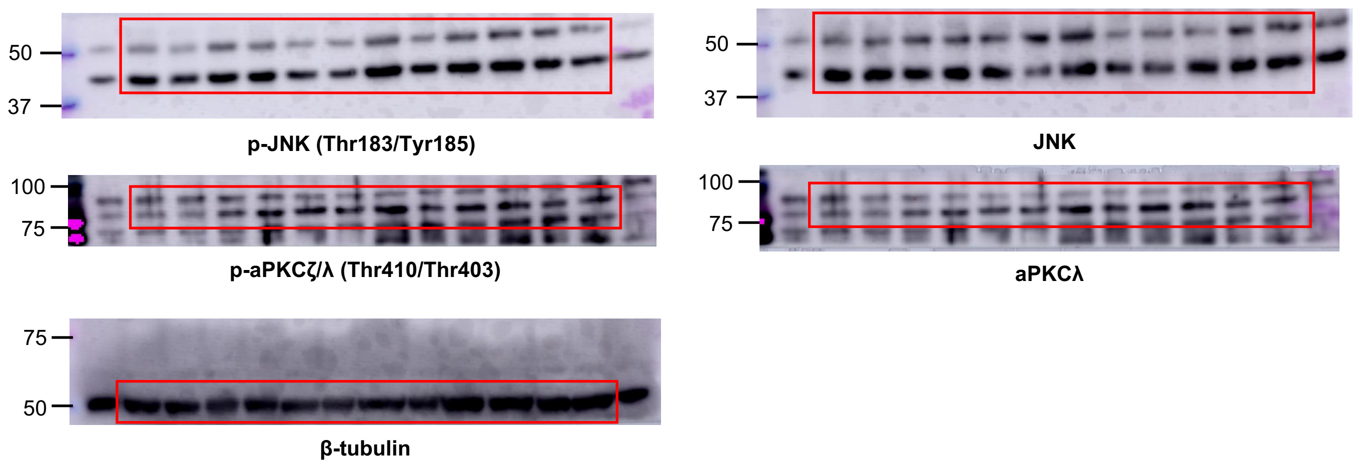

D Figure S3D

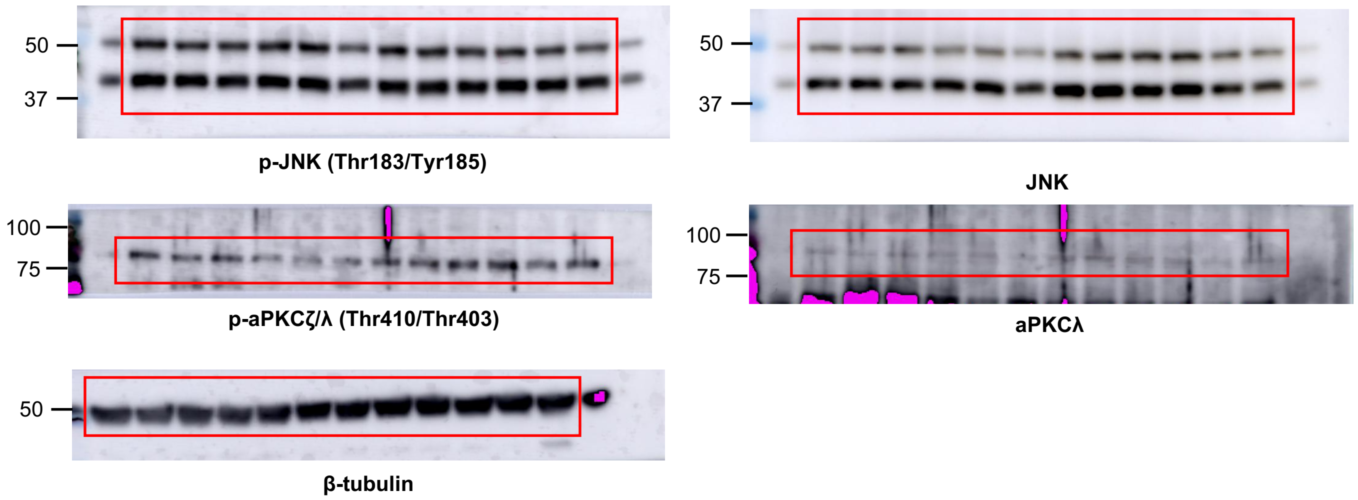

Supplementary File 5 (Figure S5) (continued)

E Figure S3E

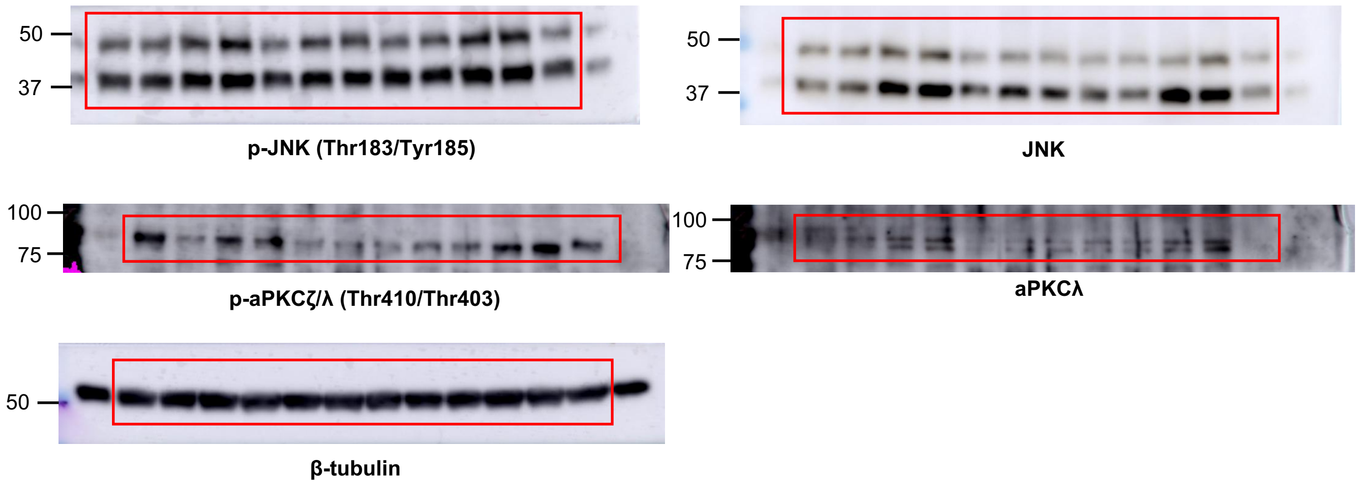

Supplementary File 5 (Figure S5). Full images of western blots. (A) Figure S3A; (B) Figure S3B; (C) Figure S3C; (D) Figure S3D; (E) Figure S3E: phosphorylated and total JNK, aPKCζ/λ, and β-tubulin.

Table S1. Summary of phosphorylated Ser residues on hippocampal IRS1 in all models

Types of models \ Phosphorylation of hippocampal IRS1 at Ser sites	mSer 307	mSer 612	mSer 632/635	mSer 1097
Middle-aged (34-36 weeks old) DIO mice	↑	N.S.	N.S.	↑
STZ (10 weeks old) mice	N.S.	N.S.	N.S.	N.S.
Aged (84 weeks old) mice	↑	↑	↑	N.S.
Young (12 weeks old) APPKI$^{NL\text{-}G\text{-}F}$ mice	↑	↑	N.S.	↑
Middle-aged (34-36 weeks old) APPKI$^{NL\text{-}G\text{-}F}$ mice	N.S.	↑	↑	↑

N.S.: no significant difference; ↑ : increased

Table S1. Summary of phosphorylated Ser residues of hippocampal IRS1 in all models.
